# An Integrated Cyber-Physical Digital Twin Architecture with Quantitative Feedback Theory Robust Control for NIS2-Aligned Industrial Robotics

**DOI:** 10.3390/s26020613

**Published:** 2026-01-16

**Authors:** Vesela Karlova-Sergieva, Boris Grasiani, Nina Nikolova

**Affiliations:** Department of Industrial Automation, Faculty Automatics, Technical University of Sofia, Blvd. Kl. Ohridski 8, 1000 Sofia, Bulgaria; ninan@tu-sofia.bg

**Keywords:** digital twin, QFT robust control, industrial robots, PLC-based control, NIS2 directive, cybersecurity, cyber-physical systems, Industry 4.0, industrial communication

## Abstract

**Highlights:**

**What are the main findings?**

- The proposed cyber-physical digital twin with QFT and NIS2 security (CPDTQN) achieves high-precision joint and Tool Center Point (TCP) tracking under parametric uncertainty, worst-case dynamics, and external disturbances.
- Secure PLC-MATLAB-ROBOGUIDE integration demonstrates real-time consistency between simulation and twin execution, with negligible latency introduced by NIS2-aligned security mechanisms.

**What are the implications of the main findings?**

- The CPDTQN architecture enables reliable deployment of robust controllers in industrial Operational Technology (OT) environments, maintaining stability even under communication delays, jitter, and cyber-resilience enforcement layers.
- Digital twin-based validation provides a safe, repeatable, and traceable framework for verifying control robustness, cyber-resilience, and regulatory (NIS2) compliance prior to physical deployment.

**Abstract:**

This article presents an integrated framework for robust control and cybersecurity of an industrial robot, combining Quantitative Feedback Theory (QFT), digital twin (DT) technology, and a programmable logic controller–based architecture aligned with the requirements of the NIS2 Directive. The study considers a five-axis industrial manipulator modeled as a set of decoupled linear single-input single-output systems subject to parametric uncertainty and external disturbances. For position control of each axis, closed-loop robust systems with QFT-based controllers and prefilters are designed, and the dynamic behavior of the system is evaluated using predefined key performance indicators (KPIs), including tracking errors in joint space and tool space, maximum error, root-mean-square error, and three-dimensional positional deviation. The proposed architecture executes robust control algorithms in the MATLAB/Simulink environment, while a programmable logic controller provides deterministic communication, time synchronization, and secure data exchange. The synchronized digital twin, implemented in the FANUC ROBOGUIDE environment, reproduces the robot’s kinematics and dynamics in real time, enabling realistic hardware-in-the-loop validation with a real programmable logic controller. This work represents one of the first architectures that simultaneously integrates robust control, real programmable logic controller-based execution, a synchronized digital twin, and NIS2-oriented mechanisms for observability and traceability. The conducted simulation and digital twin-based experimental studies under nominal and worst-case dynamic models, as well as scenarios with externally applied single-axis disturbances, demonstrate that the system maintains robustness and tracking accuracy within the prescribed performance criteria. In addition, the study analyzes how the proposed architecture supports the implementation of key NIS2 principles, including command traceability, disturbance resilience, access control, and capabilities for incident analysis and event traceability in robotic manufacturing systems.

## 1. Introduction

Industrial robots are a key component of modern manufacturing systems, where high precision, robustness, and reliability are required in the presence of varying loads, mechanical interactions, and dynamic uncertainty [1,2,3]. The accuracy and robustness of industrial manipulators become critically important in the context of increasing automation, short production cycles, and the strict cyber-resilience requirements imposed by contemporary regulatory frameworks.

The dynamics of robotic manipulators depend on configuration, inertial parameters, and friction, which makes control highly sensitive to changes in the operating environment. These dependencies complicate the achievement of guaranteed stability and performance using classical control laws based on traditional linear methods [4]. In addition, the presence of noisy measurements and model uncertainty necessitates optimal and robust estimation of states and parameters [5]. The challenges become even more pronounced in industrial architectures based on programmable logic controllers (PLC), where scan-cycle frequency, communication delay, and network jitter directly affect control performance [6,7].

Quantitative feedback theory offers a systematic approach to robust control by formulating design requirements through frequency-domain bounds and plant templates that cover the admissible parameter variations [8,9,10]. The methodology is suitable for multi-axis robots whose dynamics vary with configuration. However, full dynamic decoupling through inverse dynamics requires accurate parameterization and significant computational resources, making it difficult to implement in real time in PLC-based systems [2,11]. This motivates the use of a partially decoupled approach in which the dominant nonlinearities are compensated, while the remaining cross-couplings are treated as structured uncertainty—an idea rooted in the classical works of Luh, Spong, and Vidyasagar [2,11] and later adapted to QFT by Boje [12]. A consolidated classification of digital twin approaches and trends in their development is presented in [13].

In parallel with advances in robust control methods, the digital twin has emerged as a key tool for virtual verification, simulation of extreme operating regimes, and risk analysis in industrial environments [14,15,16]. Platforms such as ROBOGUIDE enable detailed modeling of the kinematics and dynamics of industrial robots and support communication with real PLCs. Such PLC-in-the-loop digital architectures make it possible to evaluate real effects of communication delays, disturbances, and worst-case dynamic models without risk to physical equipment [17].

Recent papers investigate PLC-based digital twin architectures for virtual commissioning, supervision, and monitoring of cyber-physical systems, including realistic simulation of communication and execution constraints [18,19]. However, in these works, the digital twin is typically used for logic verification or standard controllers, without formal synthesis of robust control under parametric uncertainty and without analysis of worst-case dynamics in the control loop.

This highlights a gap between contemporary digital twin implementations and robust control methods applied in real industrial PLC environments.

For example, in [20], a digital twin model for virtual commissioning is developed and validated, including verification of PLC control code and data exchange between software platforms, whereas in [21] a digital twin-enhanced supervisory system is demonstrated with a real deployed implementation in a cyber-physical energy environment, but without formal robust-control synthesis and without a worst-case dynamics analysis within the control loop.

A further need for an integrated, robust, and cyber-resilient architecture arises from the NIS2 Directive, which imposes requirements on industrial operators for command traceability, access control, observability, and resilience during cyber incidents [22,23], as well as from recent research in the fields of industrial cybersecurity and cyber-physical system resilience [24,25]. This transforms the PLC from a mere execution controller into a security gateway and necessitates an architecture that simultaneously maintains high control accuracy and enhanced cyber-resilience.

### 1.1. Motivation

Despite the significant progress in robust control, digital twin technologies, and industrial automation, there is still a lack of an integrated framework that simultaneously applies QFT for controlling a multi-axis industrial robot with parametric uncertainty, validates the behavior through a realistic PLC-in-the-loop twin architecture, incorporates the NIS2 principles of observability, traceability, and resilience, and operates under real industrial communication and PLC cycle constraints.

Existing implementations of QFT in robotics often rely on highly simplified models and do not consider real network effects or PLC limitations [8,9,10]. Conversely, digital twin systems typically depend on standard Proportional–Integral–Derivative (PID) or vendor-specific controllers and are rarely used for formal verification of robust control strategies [14,15,16]. Finally, most architectures oriented towards cyber-resilience and NIS2 focus on network security but do not include an analysis of the control algorithms themselves [22,23].

These gaps motivate the development of a digital twin with QFT and NIS2 Security framework that combines robust control, realistic virtual execution, and cyber-resilience aligned with industrial standards.

**Figure 1:**
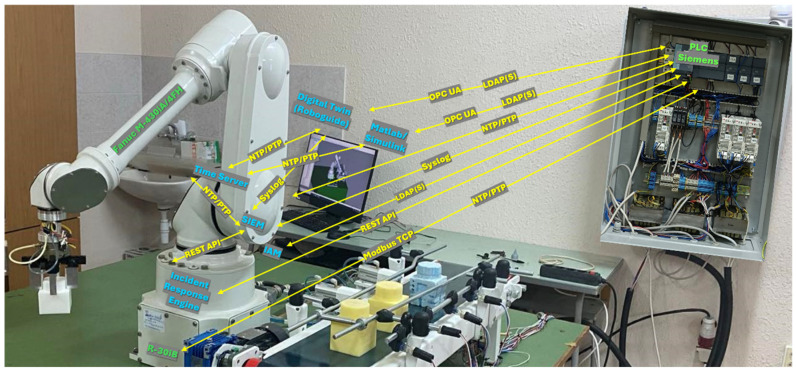
Experimental platform of the cyber-physical digital twin with QFT and NIS2 security (CPDTQN).

In this work, these requirements are addressed through an experimental platform—the cyber-physical digital twin with QFT and NIS2 security CPDTQN, shown in Figure 1—which integrates an industrial robot, a Siemens PLC, and a digital twin in the ROBOGUIDE environment, interconnected through standard industrial protocols.

In Figure 1, the green labels denote the physical devices (industrial robot, Siemens PLC, sensors and actuators), the blue labels denote the virtual services deployed on the industrial server (digital twin, MATLAB/Simulink R13, Security Information and Event Management (SIEM), incident response engine), and the yellow labels represent the communication protocols between components (Modbus Transmission Control Protocol/Internet Protocol (TCP/IP), Open Platform Communications Unified Architecture (OPC UA), Network Time Protocol/Precision Time Protocol (NTP/PTP), REST API (Representational State Transfer Application Programming Interface), Syslog (System Logging Protocol), LDAP (Lightweight Directory Access Protocol)).

### 1.2. Research Questions and Main Contributions

Research Questions.

The present study is aimed at addressing the following research questions:

(RQ1) How can an integrated cyber-physical architecture be designed that combines robust control, PLC-based real-time execution, and a synchronized digital twin, while simultaneously ensuring observability and traceability in accordance with the requirements of the NIS2 Directive?

(RQ2) To what extent can a partially decoupled QFT approach guarantee robust tracking and stability under parametric uncertainty, worst-case dynamics, and external disturbances in a multi-axis industrial robot implemented in a PLC environment?

(RQ3) What is the impact of communication, synchronization, and security mechanisms on the accuracy and robustness of the closed-loop system during twin-based validation of the control strategy?

These research questions are addressed through a research plan systematically presented in the paper, which includes system modeling, synthesis of QFT controllers and prefilters, PLC-based implementation, synchronization with a digital twin, and experimental validation using quantitative performance indicators.

The main contributions of the present work are as follows:

(1) Robust QFT control of a five-axis industrial manipulator. A control-oriented model of the servo axes of the FANUC M-430iA/4FH is developed, on which QFT controllers and prefilters are synthesized, ensuring robust tracking under parametric uncertainty, external disturbances, and varying loads. This extends existing QFT applications, which typically consider only simulation environments.
(2) Partially decoupled multi-axis QFT formulation. A methodology is presented in which the dominant nonlinearities are compensated, while the residual interactions are treated as structured uncertainty, enabling synthesis via standard QFT bounds without requiring full dynamic inversion—a rarely applied approach in the literature.
(3) Twin-based validation through CPDTQN architecture. An integrated MATLAB-PLC-ROBOGUIDE system with Modbus TCP, OPC UA, and PTP synchronization is implemented, enabling evaluation of the controller under conditions close to real industrial environments, including communication delays, PLC cycle timing, and worst-case dynamic models. This combination of QFT control, PLC-in-the-loop execution, and a synchronized digital twin is practically absent from prior research.
(4) NIS2-oriented cyber-resilient architecture. The PLC is treated not merely as an execution device but as a protected zero-trust gateway with transport layer security (TLS) protection, X.509 authentication, CRC verification, Write Once, Read Many (WORM)-based logging, and forensic analysis capabilities. This augments the digital twin architecture with mechanisms for traceability, observability, and security in line with NIS2.
(5) Quantitative twin-based evaluation via root mean square error (RMS), maximum, and 3D TCP error. Unified metrics are introduced for comparing MATLAB/QFT behavior with twin-based execution, including nominal, worst-case, and disturbed scenarios. The results demonstrate close alignment between simulation and twin execution, as well as the absence of negative impact from the security mechanisms, representing a novel contribution to the cyber-resilience analysis of robotic systems.

### 1.3. Structure of the Paper

The structure of the paper reflects the research plan, which positions the proposed study with respect to the state of the art and logically connects the architectural design, robust control methods, and experimental validation with the formulated research questions.

The paper is organized as follows. Section 2 presents a review of related work in robust control, digital twin technologies, and cyber-resilience of industrial systems. Section 3 describes the proposed CPDTQN architecture and the communication mechanisms employed. Section 4 introduces the kinematic and dynamic models of the robot, as well as the parameter uncertainties used in the QFT analysis. Section 5 formulates the NIS2-oriented aspects, including security, traceability, and their integration into the CPDTQN architecture. Section 6 presents simulation results, dynamic analysis, and twin-based validation. Section 7 provides a discussion and interpretation of the results. Section 8 summarizes the main conclusions and outlines directions for future work.

## 2. Related Work

### 2.1. Robust Control, Digital Twins, and Industrial Communication

Modern industrial robotic systems are evolving toward deep integration between the physical installation, digital models, and secured communication layers—a trend characteristic of Industry 4.0 and Industry 5.0 environments [26,27,28,29].

However, the transition to real-time cyber-physical architecture remains only partially realized. Digital twin technologies have become an essential tool for diagnostics and simulation, yet many existing developments remain predominantly offline- or simulation-oriented rather than integrated into real closed-loop robotic systems [30,31,32,33].

In robotics, DT models enable trajectory validation and anomaly analysis, but most solutions lack real-time coupling with industrial controllers, which limits their applicability under dynamic disturbances and uncertainty [34,35].

From a control perspective, robust methods-including QFT-provide a well-established mechanism for handling parametric uncertainty [8,9,10,11,36]. Despite this strong theoretical foundation, few studies examine the application of QFT control in industrial settings with real communication constraints such as PLC scan cycles, latency, or jitter. Even works addressing dynamic or uncertain manipulators [10,12,37] rarely establish an explicit connection between robust control and digital twins, which significantly restricts the validation of control strategies in realistic scenarios.

Some studies combine robust control with DT-based verification, but these developments are fragmented and do not consider key industrial factors such as network load, communication protocols, and synchronization [32,38].

### 2.2. Research Gaps and Positioning of the Present Work

Despite the substantial body of literature on robust control, digital twin technologies, and industrial cybersecurity, the analysis reveals several significant, systematic, and consistently overlooked gaps.

First, there is an almost complete absence of studies that combine QFT control, PLC-based closed-loop execution, and DT synchronization under realistic communication conditions such as latency, jitter, and packet loss [32,38,39]. Existing developments typically consider only isolated components—QFT control without a DT, DT without PLC-in-the-loop capability, or PLC implementations without robust control strategies—making the results unsuitable for deployment in industrial environments.

Despite the progress in digital twin systems with PLC integration and real-world deployments [20,21], these solutions do not address robust control under parametric uncertainty, nor do they quantitatively assess the impact of PLC cyclic operation, communication latency, and security mechanisms on control performance.

Second, cybersecurity in the context of robotic systems is treated in a fragmented manner. The literature on NIS2 provides a mature framework for risk management [22,23,40,41,42], but it is not integrated with control algorithms, and even less so with real PLC architectures. There is a lack of publications examining the impact of cryptography, RBAC, X.509 authentication, WORM logging, and SIEM correlation on the timing dynamics of the control process.

Third, there is no quantitative link between robust control strategies and regulatory requirements for resilience, accountability, and traceability. Although QFT is well studied for handling uncertainty [8,9,10,11,12,13,14,15,16,17,18,19,20,21,22,23,24,25,26,27,28,29,30,31,32,33,34,35,36,37,38,39,40,41,42,43], its behavior within a cyber-resilient, regulation-aligned architecture remains insufficiently explored.

The present work addresses these gaps by proposing an integrated CPDTQN architecture that accomplishes the following:

- Incorporates multiple uncertainty models [10,12,36,37],
- Treats the PLC as a deterministic security gateway [18,19,20,21,39,44,45,46,47,48,49],
- Integrates a bidirectional DT for real-time diagnostics [30,31,32,33,34,35],
- Applies regulatory NIS2-compliant mechanisms [22,23,40,41,42].

Table 1 summarizes these gaps and clearly illustrates the lack of overlap between the existing research domains.

**Figure 2:**
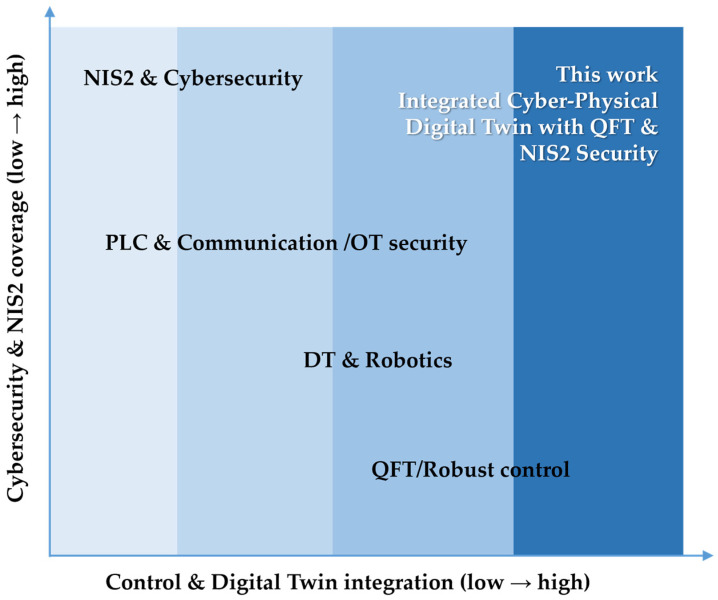
Literature gap map showing the position of the proposed integrated CPDTQN architecture relative to existing research clusters in robust control, digital twins, industrial communication, and cybersecurity.

Figure 2 additionally visualizes the missing links between the four key components: QFT, PLC, digital twin, and NIS2 security, highlighting the need for an entirely new integrated framework.

Figure 2 and Table 1 summarize the positioning of the present study with respect to the existing and reviewed literature. From research in the fields of QFT and robust control, the principles of worst-case analysis, parametric uncertainty, and frequency-domain controller synthesis are adopted. From digital twin studies, the concepts of virtual validation, simulation of realistic operating scenarios, and PLC-in-the-loop execution are utilized. The literature on PLCs and industrial communications contributes established models for cyclic operation, synchronization, and communication protocols. Research in the areas of cybersecurity and NIS2 provides the framework for requirements related to traceability, observability, and resilience.

The present work builds upon these directions through their simultaneous integration into a unified cyber-physical CPDTQN architecture, in which QFT control is executed and validated in a realistic PLC-based environment with a synchronized digital twin and a quantitative analysis of the impact of communication and security mechanisms on control performance, a combination that is absent from the existing literature. Thus, the present study positions itself at the unique intersection of robust control, industrial cyber-physical integration, and regulatory cyber-resilience, addressing a consistently overlooked gap in contemporary literature.

## 3. CPDTQN Architecture for the FANUC M-430iA/4FH Robot

This section presents the CPDTQN architecture, which integrates the digital twin [44] of the industrial robot FANUC M-430iA/4FH (FANUC Bulgaria EOOD, Sofia, Bulgaria), a Siemens PLC controller (Siemens EOOD, Sofia, Bulgaria), and MATLAB/Simulink-based QFT regulators. The CPDTQN architecture combines the physical work cell, the virtual model in ROBOGUIDE V9, and the algorithmic control layer, maintaining synchronized bidirectional data exchange, real-time execution, and NIS2-compliant mechanisms for monitoring and traceability [55]. This organizational structure provides the environment required for validating the kinematic and dynamic models introduced in Section 4, as well as for conducting the twin-based experimental analyses presented in Section 6.

The digital twin and its associated services (MATLAB/Simulink, SIEM, Identity and Access Management (IAM), and the incident-response module) are executed on a dedicated industrial server that is logically segmented from the corporate Information Technology (IT) infrastructure. The physical devices, the robot, the PLC, and the peripheral mechanisms are placed in an isolated OT zone. This multilayer segmentation meets the NIS2 requirements for minimizing the exposed attack surface, ensuring traceability, and separating critical assets [55].

Communication between components is implemented through industrial Ethernet-based protocols. Modbus TCP/IP serves as the primary channel for transmitting joint reference values and telemetry between the PLC and the robot. Structured data, diagnostic information, and synchronization events are exchanged through PLC-based mechanisms, including OPC UA secure channels, NTP/PTP time alignment, and protected logging. MATLAB/Simulink executes the QFT-based controller with a fixed sampling period of $T_{s}=20\;\mathrm{ms}$ and sends control values to the PLC, which validates, routes, and forwards the data to both the robot and the digital twin [49].

In this way, the CPDTQN architecture maintains real-virtual coherence, enables evaluation of latency, jitter, and disturbances, and provides NIS2-compliant traceability of all control actions, log entries, and system events, as elaborated in Section 5.

### 3.1. Digital Twin Concept and Objectives

The digital twin of the FANUC M-430iA/4FH represents a dynamic and synchronized virtual replica of the physical manipulator [44,46,53], maintaining bidirectional data exchange with the Siemens PLC and MATLAB. The requirements for kinematic and dynamic modeling presented in Section 4 are followed, providing an environment for verification of QFT-based control. The main objectives of the digital twin are as follows:

1. Accurate virtual modeling. The twin uses a 3D model with hierarchical links, a defined TCP, and coordinate frames. Alignment with the physical robot is ensured through transformation matrices between ROBOGUIDE, the PLC, and MATLAB, analogous to the approaches described in Section 4.
2. Real-time synchronization. The data exchange includes joint angles, statuses, and diagnostic registers at frequencies of 20–50 Hz for motion-critical signals and 50–200 Hz for telemetry. These values are consistent with the PLC scan cycle and the communication constraints of the industrial infrastructure.
3. Support for QFT-based control. The digital twin receives trajectories, inverse kinematics (IK) is computed in MATLAB, movement is visualized, and measurements are returned, enabling comparison between nominal and worst-case models. This supports robustness analysis under disturbances.
4. Reduction in risk and setup time. ROBOGUIDE enables validation of trajectories, collision scenarios, end-effector positioning, and control strategies without interrupting the production process.
5. NIS2-compliant observability. The digital twin maintains NTP/PTP synchronization, centralized logging, and IAM integration, ensuring traceability and access control.

### 3.2. Communication Architecture Between the Robot and the Digital Twin

**Figure 3:**
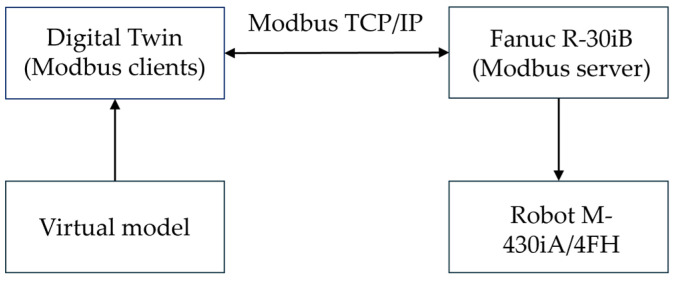
Communication overview diagram.

Communication between the industrial robot FANUC M-430iA/4FH and its digital twin is implemented through industrial Ethernet-based protocols that enable bidirectional real-time data exchange. This functionality is available in ROBOGUIDE version 8.0 and above, which supports external communication between the virtual and physical R-30iB robot controllers. The overall communication scheme is illustrated in Figure 3, showing the connection between the M-430iA/4FH robot and its digital twin, as well as the primary Modbus TCP/IP channels.

The digital twin executed in ROBOGUIDE interacts with the robot controller via Modbus TCP/IP; within the proposed architecture, a secure implementation of the protocol (Modbus TCP with TLS), commonly referred to as Secure Modbus, is used.

This communication protocol is used for the exchange of control commands, telemetry, and status information. The data exchange uses Modbus TCP/IP (IEC 61158), with the FANUC R-30iB controller operating as a Modbus Server and the digital twin functioning as a Modbus Client. The characteristics of the Modbus TCP communication channel are summarized in Table 2.

The Modbus exchange includes coils, discrete inputs, holding registers, and input registers, through which joint positions, logical states, diagnostic parameters, alarms, and trajectory information are transmitted. The overall communication flow between MATLAB, the PLC, the robot, and the digital twin encompasses stages of initialization, state synchronization, and the command phase (IK and QFT control, execution and visualization in ROBOGUIDE, diagnostics, and safety). A representative register mapping for robot feedback and command exchange is shown in Table 3.

**Figure 4:**
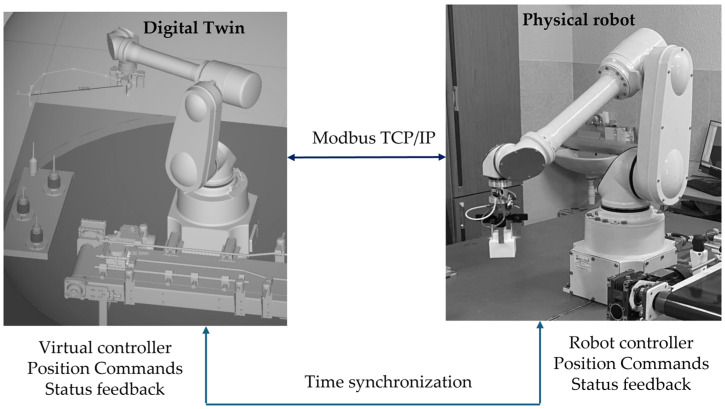
Timing and synchronization diagram.

Synchronization of the execution time between the PLC, MATLAB, and ROBOGUIDE is critical for comparing real-virtual trajectories. NTP and PTP are used, and the overall scheme is shown in Figure 4. This synchronization enables the correction of communication delays τ when evaluating trajectory-tracking errors.

Due to the differences between the coordinate systems of ROBOGUIDE and the physical controller, transformation matrices are used to align the TCP, zeroing positions, and kinematic frames, ensuring compatibility with the joint dynamic models derived in Section 4. The communication link between the digital twin and the real robot provides reduced setup time, detection of latency, jitter, and disturbances, as well as the capability for integration into Industry 4.0 environments.

The limitations of this communication include sensitivity to inaccurate model-parameter identification, inherent latency, and the non-deterministic nature of Modbus TCP, as well as the need for periodic updates of the DT model. In the proposed CPDTQN architecture, these limitations are mitigated through PLC-based cyclic operation, fixed control periods, and a clear separation between motion-critical and non-critical communication flows, enabling the closed-loop behavior to remain predictable.

### 3.3. Integrated CPDTQN Architecture with Security Extensions

**Figure 5:**
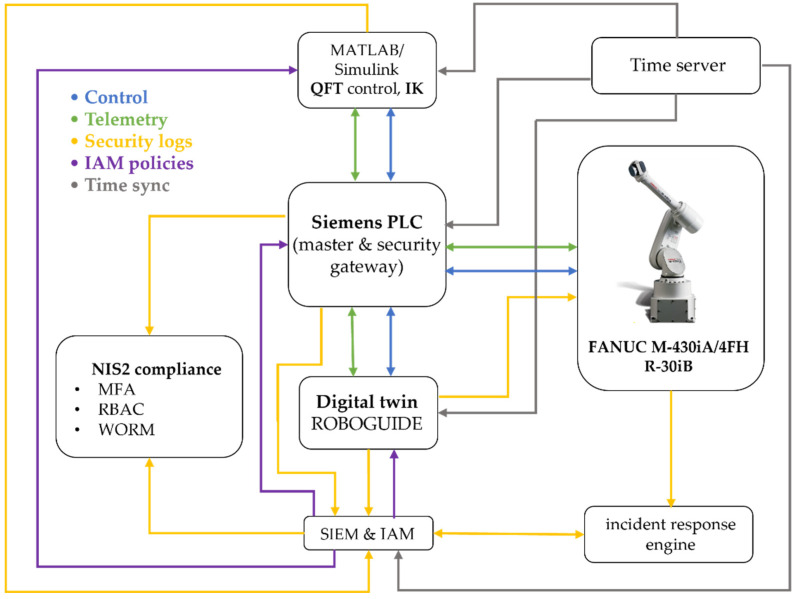
Architecture of the cyber-physical digital twin with QFT and NIS2 security (CPDTQN).

The integrated CPDTQN architecture unifies control, simulation, and observability of the FANUC M-430iA/4FH industrial robot within a single cyber-physical framework [39,46,51]. The overall architectural layout is shown in Figure 5, which extends both the basic digital twin concept presented in Figure 4 and the conceptual model illustrated in Figure 1.

The CPDTQN architecture shown in Figure 5 illustrates the various communication flows and functions, which are color-coded. The first flow is the Control flow, shown in blue. Here, control commands are exchanged between the MATLAB/QFT controller, the Siemens PLC, and the digital twin in ROBOGUIDE. The PLC receives control inputs from MATLAB over OPC UA (TLS 1.3, X.509) [49], validates them, and forwards them to the robot via Modbus TCP [48,50], while the digital twin uses these commands to simulate the motion and validate the trajectories.

OPC UA was selected not only because of its built-in security mechanisms, but also because it is a widely adopted communication standard in Industry 4.0, used for reliable communication and connectivity between diverse industrial systems, as well as for integration across control, supervisory, and information functions. An additional advantage of OPC UA is its ability to provide structured representations of data and events, which makes it suitable for digital twin synchronization and NIS2-oriented monitoring.

The second flow is Telemetry, shown in green. It represents the stream of telemetry data from the real FANUC M-430iA/4FH robot and the R-30iB controller to the PLC and the digital twin. This includes joint positions, status signals, alarms, and diagnostic data, which enable real-time synchronization and monitoring.

The third flow is Security logs, shown in yellow. It carries log events to the SIEM for analysis and correlation, including monitoring of command operations, CRCs, alarms, and system states.

Identity and role management via LDAP/Kerberos/OAuth2, the application of Role-Based Access Control (RBAC) policies, and access control to the PLC, the digital twin, and the MATLAB/QFT controller are implemented through the IAM policies communication flow, shown in purple in Figure 5.

Time synchronization via NTP/PTP across all components, providing consistent timestamps for traceability, latency, and jitter correction, and digital forensic analysis implemented through the Time sync flows, shown in gray.

Functionally, the CPDTQN architecture in Figure 5 consists of three layers:

- Physical Layer contains the real FANUC M-430iA/4FH robot, the R-30iB controller, and the peripheral infrastructure. The robot operates in Modbus Server mode and transmits telemetry to the PLC, which in turn sends reference setpoints and diagnostic data to MATLAB and ROBOGUIDE.
- Virtual Layer, executed in ROBOGUIDE and MATLAB/Simulink, includes the digital twin, the QFT controller [45], and the additional NIS2-related services (SIEM, IAM, Incident response engine). It is logically isolated from the office IT environment in accordance with the segmentation requirements outlined in Section 5.
- Coordination Layer (PLC Gateway) synchronizes all communication channels and acts as both the master controller and a security gateway. The PLC receives QFT-based control values from MATLAB, validates them, and forwards them to the robot via Modbus TCP/IP, while telemetry from the robot is returned to the PLC and routed to the digital twin. In this way, a closed-loop system is maintained that accounts for latency, jitters, and external disturbances.

In Table 4, the communication links between the PLC, the robot, MATLAB/Simulink, the digital twin, and the NIS2-compliant services are summarized, showing the role of each protocol in coordinating the physical and virtual systems. The matrix demonstrates how the PLC functions simultaneously as a master controller and a security gateway, integrating control commands, telemetry, logs, and synchronization channels into a unified communication infrastructure.

Through time synchronization, centralized command validation, and real-virtual consistency, the CPDTQN architecture turns the digital twin into an active component of the control loop. This enables verification of the QFT controllers under realistic communication conditions, latency, and disturbances, and provides a platform for cyber-resilient control aligned with the requirements of NIS2.

### 3.4. NIS2-Compliant Security, Logging, and Traceability

In accordance with the requirements of Directive (EU) 2022/2555 (NIS2), the digital twin architecture is augmented with mechanisms for authentication, access control, cryptographic protection, centralized logging, and event traceability. These functions ensure that the operation of the FANUC M-430iA/4FH industrial robot complies with regulatory standards for the protection of critical cyber-physical systems.

Access to the PLC, the digital twin, and the MATLAB/QFT controller is governed by a multilayer authentication scheme that includes multi-factor authentication (MFA), X.509 certificates for establishing TLS 1.3 sessions, and role-based access control (RBAC), under which operators, engineers, and automated services receive strictly defined permissions. Identity management is performed centrally through IAM services (LDAP/Kerberos/OAuth2), and all successful and failed access attempts are recorded in an immutable database, ensuring full audit traceability in the spirit of NIS2.

To secure communication between the physical and virtual parts of the system, a range of cryptographic and network mechanisms are applied: TLS 1.3 encryption for OPC UA channels, Internet Protocol Security (IPsec) tunneling for Modbus TCP/IP traffic, network segmentation via Virtual Local Area Networks (VLANs) to separate control, telemetry, and administrative services, as well as deep packet inspection (DPI) within the PLC to detect malicious or malformed packets. These measures mitigate the risk of unauthorized commands, data manipulation, and man-in-the-middle attacks.

The centralized logging system aggregates logs from the PLC, ROBOGUIDE, and MATLAB via Syslog over TLS. Logged events include control commands, Modbus operations, alarms, diagnostic parameters, TCP and joint deviations, and IAM events (login/logout, role changes). All records are stored in WORM format, ensuring an immutable audit trail and fulfilling NIS2 requirements for transparency and accountability.

Precise temporal correlation between the physical robot, PLC, and digital twin is achieved through the Network Time Protocol (NTP) and the Precision Time Protocol (PTP). A unified time base enables correction of latency, jitter, and Round-Trip Time (RTT) when analyzing tracking errors and facilitates forensic investigation in which SIEM events are correlated using a universal timestamp.

Integration with the SIEM enables automated incident monitoring. The system detects invalid CRC values, unauthorized access attempts, motion anomalies (e.g., deviations in the TCP trajectory), excessive temperatures, or inconsistencies in speed and position. Upon detecting an anomaly, the SIEM generates an alert to the incident response engine, which may block a command, temporarily isolate a device via PLC rules, or trigger a safety mode (freeze motion). All actions are recorded for forensic analysis.

The proposed architecture supports complete traceability through synchronized timestamps, cryptographically signed WORM logs, and automatic correlation between real and virtual states. Each command pathway (MATLAB → PLC → FANUC M-430iA/4FH → PLC → digital twin → SIEM) can be reconstructed with high precision, enabling detailed digital forensic analysis. These mechanisms satisfy all key NIS2 requirements for protection, observability, accountability, and resilience of critical cyber-physical systems, making the CPDTQN architecture a reliable platform for industrial validation under real cyber-risk conditions.

### 3.5. Functional Roles of System Components

The integrated CPDTQN architecture comprises several interconnected components [46] that collectively realize control, synchronization, monitoring, and traceability of the FANUC M-430iA/4FH industrial robot. As shown in Figure 1 and Figure 5, communication between these elements is accomplished through a multichannel industrial infrastructure centrally coordinated by Siemens PLC. The system maintains a closed loop of control, feedback, and virtual verification, and the primary links between components and protocols are summarized in Table 4.

- Siemens PLC acts as the central coordination module, functioning simultaneously as the master controller and security gateway. As a controller, it exchanges control values and telemetry with the robot via Modbus TCP/IP (details in Table 2 and Table 3), supports polling cycles of 20–50 ms for motion-critical signals, and distributes information to MATLAB/Simulink and ROBOGUIDE. As a security gateway, it enforces authentication via X.509 certificates, encrypts OPC UA sessions, filters traffic via Deep Packet Inspection (DPI), and applies RBAC/IAM policies in accordance with NIS2. Its central role in communication flow is clearly depicted in Figure 1.
- MATLAB/Simulink implements the algorithmic layer of control, performing inverse IK, the QFT controllers for all five axes, and robustness analysis under nominal and worst-case models (as detailed in Section 4). Data exchange with the PLC is conducted through a secure OPC UA channel using the standard Simulink OPC UA Read/Write blocks. Telemetry registers from the PLC are updated every control cycle directly into the Simulink model, while the computed control values are written back into the PLC’s OPC UA address space—never bypassing the PLC gateway. This ensures that the QFT controller operates synchronously with the PLC polling cycle and maintains the NIS2-compliant system structure. Simulink also performs continuous tracking-error evaluation, RMS metrics, and disturbance sensitivity assessment, forwarding these data to the SIEM for observability and traceability. The combination of IK, QFT control, and OPC UA synchronization enables MATLAB to function as the central algorithmic node that maintains coherent real-virtual behavior between the physical robot and the digital twin.
- The digital twin, implemented in ROBOGUIDE, is a high-fidelity virtual replica of the robot [44], reproducing its kinematics and dynamics in real time. It visualizes the trajectories generated by MATLAB and the PLC and enables real-virtual comparison through the synchronization concept shown in Figure 4. The digital twin performs simulation-based diagnostics, detects collisions, workspace limitations, and TCP anomalies, and exchanges structured data with the PLC via OPC UA (Table 4). It serves as a reference model that provides NIS2-aligned traceability and allows verification of commands prior to physical execution.
- The physical robot FANUC M-430iA/4FH and the R-30iB controller execute the control commands generated by the algorithmic layer and transmit telemetry to the PLC, including joint positions, alarms, logical states, and diagnostic parameters (see Table 3). The robot operates as a Modbus TCP server and uses NTP/PTP synchronization to maintain a unified time base with the virtual model, supporting accurate real-virtual motion analysis as shown in Figure 4.
- The SIEM system aggregates Syslog/TLS events from the PLC, MATLAB, ROBOGUIDE, and IAM, correlates them using synchronized timestamps, and analyzes deviations such as malformed command frames, CRC errors, nonstandard Modbus packets, or motion anomalies. This fulfills NIS2 requirements for monitoring, accountability, and incident-data retention and is a key element in the communication scheme shown in Figure 1.
- The incident response engine uses SIEM alerts to trigger automated actions such as terminating active sessions, restricting control commands, isolating communication channels, or activating safety modes (freeze motion or safe stop). It records all actions in a WORM log, supporting forensic analysis and ensuring operational resilience under disturbances or cyber events.
- The time server provides a unified time base for the PLC, MATLAB, ROBOGUIDE, SIEM, and the incident response engine via NTP for system time and PTP for precision synchronization. This minimizes phase offsets in real-virtual comparisons, ensures consistent timestamping (critical for NIS2 traceability), and enables correction of communication delays during the tracking analyses in Section 6.

The functional integration of all components from Figure 1 and Figure 5 and Table 4 creates a resilient and traceable cyber-physical system that supports real-time QFT control of an industrial robot, ensures reliable synchronization between the physical and virtual models, guarantees encrypted and segmented communication, and maintains a complete audit trail consistent with NIS2. This architecture provides a reliable platform for validating control strategies under realistic network conditions, latency, and disturbances, forming a comprehensive model for cyber-resilient robotic systems.

Within the proposed CPDTQN architecture, computational tasks are distributed between offline processing and real-time execution. The synthesis of the QFT controllers and prefilters is performed offline in MATLAB. The robot control loop employs linear controllers that are discretized and have fixed parameters. The PLC does not participate in controller design and does not execute computationally intensive algorithms; instead, it handles data exchange, time synchronization, and correctness and security checks. The communication and security mechanisms are integrated so as not to affect the 20 ms control cycle. The digital twin operates outside the critical control loop and does not introduce additional latency. In this way, the computational complexity within the real-time loop is reduced to the digital execution of low-order linear controllers, which is compatible with a PLC-based real-time implementation.

## 4. Control of the Multi-Axis Manipulator

### 4.1. Motivation for the QFT Approach

In the control of industrial robots, a fundamental challenge is the presence of parametric uncertainty and nonlinear interactions between mechanical links. In real manufacturing environments, the dynamic characteristics of the manipulator vary depending on load, friction, geometric configuration, and changes in the center of mass associated with different end-effectors. These factors lead to deviations between the nominal mathematical model and the real system, which may compromise control stability if the controller is designed using a conventional control law.

Quantitative Feedback Theory, introduced by Horowitz [8], provides an alternative that enables explicit quantitative handling of uncertainty. Unlike $H_{\infty }$ or μ syntheses, which require accurate matrix models, QFT uses a graphical design procedure in the Nichols plane, where the influence of parameter variations on stability and performance can be visualized. This allows the engineer to synthesize a controller that guarantees both robustness and the desired dynamic behavior for all admissible parameter variations in the system.

QFT is particularly suitable for robotic systems with multiple independent axes, where partial dynamic decoupling enables each loop to be treated as a SISO (Single Input, Single Output) system. Each regulator can therefore be designed independently, without requiring full MIMO optimization. This approach is effective when cross-axis couplings are relatively weak and can be treated as part of the structured uncertainty of the plant, represented through templates.

An additional advantage of QFT is the ability to directly define performance bounds on stability, tracking, and disturbance rejection. This is especially valuable in industrial contexts, where not only accuracy but also reliability under process variations must be ensured.

Consequently, QFT provides a clear and quantitative framework for synthesizing controllers that can be verified both in MATLAB and through the digital twin in the ROBOGUIDE environment. This makes QFT particularly suitable for integration into the proposed CPDTQN architecture (Figure 5), in which robustness and cybersecurity are treated as interconnected aspects of the control problem.

### 4.2. Hierarchical Architecture, Forward and Inverse Kinematics [3]

The control structure of the industrial manipulator follows the classical two-layer hierarchy established in robotics [2], in which the high level operates in Cartesian space and generates the desired TCP trajectories, while the low level performs local joint control through feedback. Denavit-Hartenberg (DH)-parameter formalism, forward and inverse kinematics, as well as the differential relationships defined by the Jacobian, are strictly based on the standard approaches described in [1,3].

**Figure 6:**
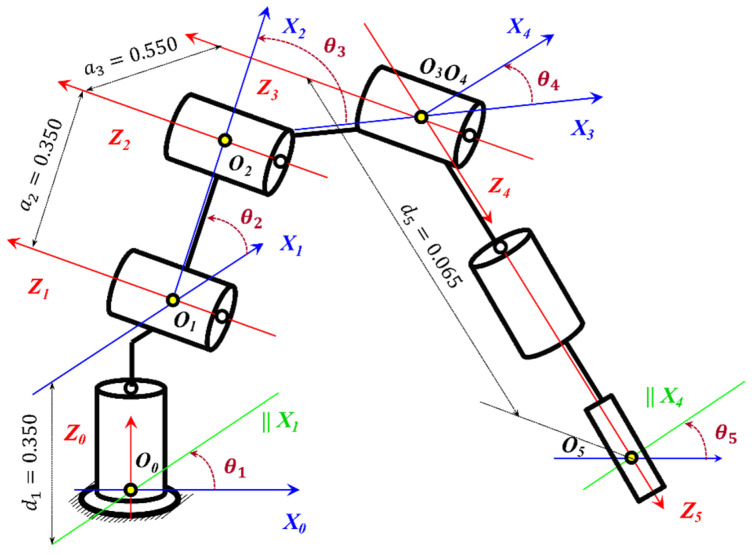
Kinematic structure and Denavit-Hartenberg coordinate assignment of the 5-DOF anthropomorphic manipulator.

This functional decomposition allows the complex nonlinear dynamic effects to be handled at the low level, whereas the high level operates on an idealized kinematic representation. The kinematic chain of the 5-DOF (Degrees of Freedom) anthropomorphic manipulator used in this study is shown in Figure 6, with a joint configuration of type R-R-R-R-R.

At the high level, the robot receives the desired end-effector trajectory in terms of position and orientation as given in (1):

(1) $$x(t)=\left[\begin{array}{c} x(t)\\ y(t)\\ z(t) \end{array}\right],\;R(t)\in SO(3)$$

where x(t) specifies the Cartesian position, and R(t) is the orientation matrix with respect to the base coordinate frame.

The objective of the high-level layer is to convert the information in (1) into reference joint coordinates through the IK given in (2):

(2) $$\theta_{ref}(t)=\left[\begin{array}{c} \theta_{1,ref}\\ \theta_{2,ref}\\ \theta_{3,ref}\\ \theta_{4,ref}\\ \theta_{5,ref} \end{array}\right]=IK\{x(t),R(t)\}.$$

Direct kinematics is obtained through the successive multiplication of the homogeneous transformations between the links (3):

(3) $$T_{0}^{5}(\theta )=T_{0}^{1}(\theta_{1})\;T_{1}^{2}(\theta_{2})\;T_{2}^{3}(\theta_{3})\;T_{3}^{4}(\theta_{4})\;T_{4}^{5}(\theta_{5}),$$

where each transformation $T_{i}^{\left(i+1\right)}$ (4) is defined by the DH parameters shown in Figure 6:

(4) $$T_{i}^{i+1}=\left[\begin{array}{cccc} \cos\theta_{i} & -sin\;\theta_{i}cos\;\alpha_{i} & sin\;\theta_{i}sin\;\alpha_{i} & a_{i}cos\;\theta_{i}\\ \sin\theta_{i} & cos\;\theta_{i}cos\;\alpha_{i} & -cos\;\theta_{i}sin\;\alpha_{i} & a_{i}sin\;\theta_{i}\\ 0 & \sin\alpha_{i} & \cos\alpha_{i} & d_{i}\\ 0 & 0 & 0 & 1 \end{array}\right].$$

The position and orientation of the end-effector (Tool Center Point, TCP) are obtained from (5):

(5) $$p(\theta )=T_{0}^{5}(1:3,4),\;R(\theta )=T_{0}^{5}(1:3,1:3).$$

The differential relationship between joint velocities and TCP velocity is expressed through the Jacobian matrix (6):

(6) $$\dot{x}=J(\theta )\dot{\theta },$$

For revolute joints, the Jacobian columns are computed using (7):

(7) $$J_{v,i}=z_{i-1}\times (p_{5}-p_{i-1}),\;J_{\omega ,i}=z_{i-1}.$$

These relationships are used to numerically refine the analytical inverse kinematics. The IK of the anthropomorphic 5-DOF manipulator is solved through the standard decomposition into two subproblems: position (axes $J_{1}–J_{3}$) and orientation (axes $J_{4}–J_{5}$). Here, *J*_1_ corresponds to the base axis, *J*_2_ to the shoulder, $J_{3}$ to the elbow, $J_{4}$ to the wrist pitch, and J_5_ to the wrist roll.

The rotation angle $\theta_{1}$ of joint $J_{1}$ is obtained from the horizontal projection (8):

(8) $$\theta_{1}=\arctan\;2\left(y,x\right).$$

The vertical and horizontal components used in (8) are given by (9):

(9) $$r=\sqrt{x^{2}+y^{2}},\;z^{\prime }=z-d_{1}.$$

The intermediate distance required for the elbow joint $J_{3}$ is computed as (10):

(10) $$D=\frac{r^{2}+{z^{\prime }}^{2}-a_{2}^{2}-a_{3}^{2}}{2a_{2}a_{3}},$$

From (10), the elbow joint angle $\theta_{3}$ follows as (11):

(11) $$\theta_{3}=arctan\;2(\pm \sqrt{1-D^{2}},\;D),$$

The shoulder joint angle $\theta_{2}$ is computed using (12):

(12) $$\theta_{2}=arctan\;2(z^{\prime },r)-arctan\;2(a_{3}sin\;\theta_{3},\;a_{2}+a_{3}cos\;\theta_{3}).$$

Once the first three joints are obtained, the orientation of link 3 is determined from the rotation matrix $R_{0}^{3}$, and the desired wrist motion is described by (13):

(13) $$R_{w}=(R_{0}^{3}{)}^{T}R\left(t\right).$$

The wrist-joint angles $\theta_{4}$ and $\theta_{5}$ are then obtained from (14) and (15):

(14) $$\theta_{4}=arctan\;2(R_{w}(2,3),\;R_{w}(1,3)),$$

(15) $$\theta_{5}=arctan\;2(R_{w}(3,2),\;R_{w}(3,1)).$$

The intermediate variable $R_{w}$ has a clear geometric interpretation and represents the radial distance, in the plane of the first two axes, between the origin of the robot coordinate system and the wrist center. It is used to simplify the geometric solution of the inverse kinematics by separating the position problem from the orientation problem, and it enables an unambiguous determination of the angles of the first two axes.

The analytical expressions obtained in (8)–(15) provide a closed-form solution to the IK. However, in the practical implementation a hybrid approach is used, combining the analytical solution with iterative Jacobian-based corrections to improve numerical robustness during fast robot trajectories or when operating near kinematic singularities.

At the low level, each of the five joints $J_{i}$ implements an independent local feedback loop (16):

(16) $$u_{i}(t)=C_{i}(s)(\theta_{ref,i}(t)-\theta_{i}(t)),$$

where $C_{i}(s)$ are the QFT controllers are synthesized with respect to nominal and uncertain dynamic models of the actuators.

The actual dynamics of the manipulator are described by the Lagrangian model (17):

(17) $$M(\theta )\ddot{\theta }+C(\theta ,\dot{\theta })\dot{\theta }+G(\theta )=u,$$

where the matrix M(θ) is dense and captures the mutual dynamic couplings among the joints $J_{i}$; $C(\theta ,\dot{\theta })$ is the matrix of centrifugal and Coriolis forces arising from the motion of the links $J_{i}$; and $G\left(\theta \right)$ is the vector of gravitational forces acting on each joint $J_{i}$ depending on the configuration.

These nonlinear interactions and external disturbances are compensated by the robust QFT controllers at a low level, enabling the high-level layer to operate on an idealized kinematic model, while the low-level control ensures the actual, robust, and accurate execution of the reference joint trajectories $\theta_{ref,i}(t)$.

### 4.3. Trajectory Generation in the Task Space [1,11]

Trajectory generation is a fundamental element of the high-level control layer, as it defines the desired motion of the tool center point (TCP) in the task space in a manner that is smooth, physically realizable, and consistent with the kinematic and mechanical constraints of the FANUC manipulator. Let the desired Cartesian trajectory be specified by a position vector and an orientation matrix as in (1). The resulting time functions must be mathematically smooth up to the second derivative to avoid abrupt changes in TCP velocity and acceleration, which could disrupt the stability of the low-level control loops.

In the present work, these requirements are satisfied through time parameterization using an optimized 7-phase S-curve profile, which ensures bounded jerk, acceleration a(t), and velocity v(t) of the desired motion trajectory s(t) of the end-effector. Let s(t)∈[0,1] denote the normalized S-curve parameter of the reference path, defined through integration of the velocity and acceleration according to (18):

(18) $$\dot{s}\left(t\right)=v\left(t\right),\;\ddot{s}\left(t\right)=a\left(t\right).$$

The acceleration a(t) follows the standard 7-phase structure (acceleration increase, constant acceleration, acceleration decrease, zero-acceleration segment, symmetric deceleration profile). The normalized position parameter s(t) serves as a universal time index, through which every point of the desired TCP spatial trajectory is reached with dynamically compatible velocity.

The desired Cartesian TCP trajectory is defined through a set of control points $P_{i}=[x_{i}\;y_{i}\;z_{i}$] between which a smooth B-spline/PCHIP interpolation is constructed. Accordingly, the final TCP position of the end-effector is obtained from (19):

(19) $$p(t)=\left[\begin{array}{c} x(s(t))\\ y(s(t))\\ z(s(t)) \end{array}\right].$$

This approach ensures $C^{2}$-class smoothness of the commanded TCP trajectory, the absence of sharp curvature transitions, and dynamic feasibility even during high-speed robot motion. The resulting trajectory p(t) is then supplied to the analytical inverse kinematics from Section 4.2, which generates the reference joint coordinates given by (20):

(20) $$\theta_{ref}(t)=IK\{x(t),R(t)\}.$$

The reference joint velocities ${\dot{\theta }}_{ref}\left(t\right)$ (21) and accelerations ${\ddot{\theta }}_{ref}(t)$ (22) are determined using the manipulator Jacobian $J^{-1}(\theta )$, as is standard for anthropomorphic robotic architectures.

(21) $${\dot{\theta }}_{ref}\left(t\right)=J^{-1}\left(\theta \right)\;\dot{x}\left(t\right)$$

(22) $${\ddot{\theta }}_{ref}(t)=J^{-1}(\theta )\;\ddot{x}(t)+{\dot{J}}^{-1}(\theta ,\dot{\theta })\;\dot{x}(t)$$

**Figure 7:**
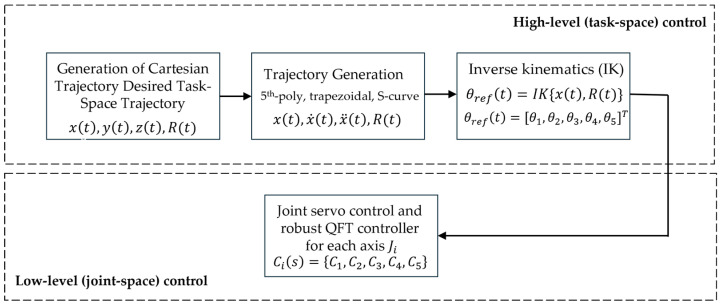
Hierarchical control architecture and inverse kinematics pipeline.

The computed expressions for the joint position $\theta_{ref}(t)$, joint velocity ${\dot{\theta }}_{ref}(t)$, and joint acceleration ${\ddot{\theta }}_{ref}(t)$ of the joints $J_{i}$ constitute the complete set of reference joint trajectories that are supplied to the low-level controller. The schematic structure of the high-level control layer is shown in Figure 7.

The high-level layer generates a trajectory in the Cartesian TCP coordinate space, which is transformed into joint reference coordinates $\theta_{ref,i}$ through IK. The low-level layer employs QFT controllers to ensure robust tracking of $\theta_{ref,i}(t)$ despite parameter variations, load changes, friction, and external disturbances. In this way, the high-level layer operates on an idealized kinematic model, while the low-level layer compensates for the actual nonlinear dynamics of the manipulator and guarantees stable and accurate execution of the motion, as illustrated in Figure 7.

**Figure 8:**
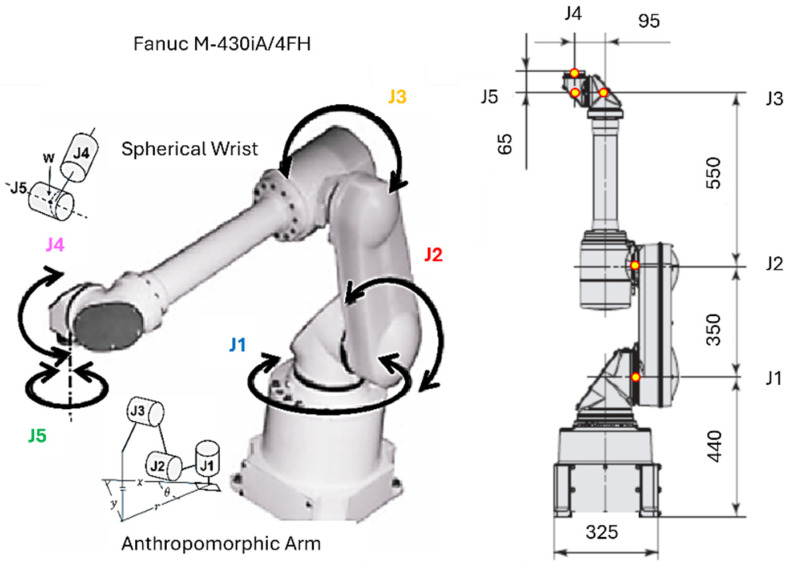
5-DOF FANUC M-430iA/4FH manipulator.

The robot used in this study is an anthropomorphic five-axis manipulator with revolute joints $J_{1},\;J_{2},\;J_{3},\;J_{4},\;J_{5}$, consisting of a shoulder, elbow, and spherical wrist, as shown in Figure 8. The position control of each joint $J_{i}$ is performed through an electric actuator, where the primary measured coordinates are the joint angular positions $\theta_{1},\;\theta_{2},\;\theta_{3},\;\theta_{4},\;\theta_{5}$. Each drive motor is modeled as a first-order element with an integrator, describing the relationship between the control input voltage $u_{i}(t)$ and the angular displacement $\theta_{i}(t)$.

In the complete dynamic model of the manipulator, cross-couplings exist between the joints $J_{i}$, arising from inertial, centrifugal, and gravitational interactions. For example, the motion of the shoulder joint $J_{2}$ with angle $\theta_{2}$ alters the inertia affecting the elbow joint $J_{3}$ and its angle $\theta_{3}$, while rotation of the base joint $J_{1}$ by angle $\theta_{1}$ can induce dynamic deviations in the wrist coordinates of joint $J_{5}$ with angle $\theta_{5}$.

These mutual interactions render the system a multi-input–multi-output (MIMO) structure, which typically requires complex matrix-based controller synthesis.

**Figure 9:**
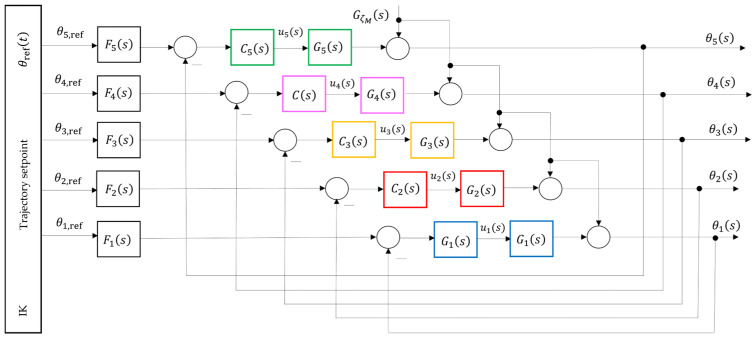
Five-axis manipulator with five independent drive motors controlled through joint coordinates $\theta_{1}-\theta_{5}$.

For the purposes of the present study, a decoupling strategy is adopted in which each joint $J_{i}$ is treated as an independent single-input-single-output (SISO) loop with its own controller $C_{i}(s)$ and plant $G_{i}(s)$, as expressed in (23) and illustrated in Figure 9.

(23) $$G_{i}\left(s\right)=\frac{\theta_{i}\left(s\right)}{u_{i}\left(s\right)}=\frac{k_{i}}{s(T_{i}s+1)},\;i=1\ldots 5$$

where $u_{i}(s)$ is the control voltage or current applied to the servo amplifier; $\theta_{i}(s)$ is the angular position of the corresponding joint $J_{i}$; $k_{i}$ is the static gain determining the relationship between the control input and the steady-state angular displacement $\theta_{i}$; $T_{i}$ is the electromechanical time constant associated with the dynamics of the servo amplifier and the effective mass moment of inertia.

### 4.4. Decoupling of the Multi-Axis Dynamics

For designing the QFT controllers, a partial decoupling approach is applied [1,2,13], which allows each joint $J_{i}$ to be treated as an independent SISO subsystem. To this end, Equation (17) is rewritten in the following form (24):

(24) $$u=M(\theta )\ddot{\theta }+d(\theta ,\dot{\theta })$$

where $d(\theta ,\dot{\theta })=C(\theta ,\dot{\theta })\dot{\theta }+G(\theta ).$

If the off-diagonal elements of the matrices M(θ) and $C(\theta ,\dot{\theta })$ are assumed to be significantly smaller than the corresponding diagonal elements, the dynamics of each joint $J_{i}$ can be approximated as a nearly decoupled SISO subsystem. Under this approximation, Equation (17) is reduced to the following scalar form (25):

(25) $$M_{ii}(\theta_{i}){\ddot{\theta }}_{i}+C_{ii}({\dot{\theta }}_{i}){\dot{\theta }}_{i}+G_{i}(\theta_{i})=u_{i}+\Delta_{i}$$

where $\Delta_{i}$ represents the aggregated effect of all cross-axis dynamic couplings (26):

(26) $$\Delta_{i}=\sum_{j\neq i}\left(M_{ij}\left(\theta \right){\ddot{\theta }}_{j}+C_{ij}\left(\theta ,\dot{\theta }\right){\dot{\theta }}_{j}\right).$$

In the context of QFT, $\Delta_{i}$ is treated as a multiplicative uncertainty (27) on the nominal plant (23):

(27) $$G_{i}(s)=G_{i0}(s)\;[1+w_{i}(s)]$$

where $G_{i0}(s)$ is the nominal dynamics of joint $J_{i}$, and $w_{i}(s)$ describes the relative deviation between the real and nominal dynamics (typically $|w_{i}(j\omega )|\leq \delta_{i}$).

Thus, each joint $J_{i}$ is controlled through the input $u_{i}(s)$ independently, following the structure in Figure 9 and Equation (28):

(28) $$u_{i}\left(s\right)=C_{i}\left(s\right)\;\left[r_{i}\left(s\right)-\theta_{i}\left(s\right)\right].$$

The interactions between the axes $J_{i}$ are accounted for in the QFT templates through an expanded range of the gains $k_{i}$ and time constants $T_{i}$ of the plant (23). These parameter ranges incorporate the effects of inertial, centrifugal, Coriolis, and gravitational cross-couplings between the joints, modeled as generalized uncertainty in each SISO subsystem.

The chosen partially decoupled approach is justified because structural independence is naturally present, each servo motor has its own actuator and encoder, enabling direct position control of $\theta_{i}$. Moreover, the electrical drives have a bandwidth significantly higher than the mechanical couplings, meaning the internal motor dynamics dominate. This is also advantageous for PLC-based synchronization, since the controller can enforce timing alignment and eliminate accumulated errors between control channels.

Consequently, the full MIMO model is reduced to five SISO loops, each with a transfer function of the form (23). These transfer functions are used in the following section to construct QFT templates and derive robust bounds. The conceptually decoupled five-axis model is shown in Figure 9, where each loop is assigned to its own QFT controller. The cross-axis interactions are not modeled as explicit dynamic couplings; instead, they are treated as multiplicative uncertainty in the transfer function of each joint $J_{i}$ as in (27). Thus, the influence of other joint motions on $J_{i}$ is encapsulated within the extended ranges of the gains $k_{i}$ and time constants $T_{i}$, reflected directly in the QFT templates.

Within the current architecture (MATLAB-PLC-FANUC M-430iA/4FH), Figure 5, a closed-loop position control of each joint $J_{i}$ is realized: the QFT controller in MATLAB computes the control input $u_{i}$ required to maintain the desired joint position $\theta_{i}$. The PLC acts as an industrial middleware layer that forwards the command to the servo drive and ensures synchronization and safety. The robot responds by adjusting the joint angle $\theta_{i}$, which is measured by the encoders and returned as feedback. Accordingly, the QFT controller governs the joint rotation $\theta_{i}$, generating torque commands that compensate for friction, load variations, and cross-coupling effects. As a result, the joint position $\theta_{i}$ of each axis remains stable and tracks the desired TCP-derived profile even under parametric variations and external disturbances, while the PLC ensures coordination between the virtual (twin) and physical system.

Partial decoupling of the manipulator dynamics and modeling each axis as a SISO system is widely used in industrial robotics and is justified by the structural independence of the drives, the presence of local encoders, and the higher bandwidth of electrical servo drives compared to the mechanical interactions between links. The main nonlinear effects, such as friction and inter-axis coupling, are not modeled explicitly; instead, structured uncertainty is introduced and reflected in the dynamic parameters of the axis models within the QFT templates. Under normal operating conditions within industrially permissible loads, this uncertainty remains covered by the adopted templates and closed-loop robustness is preserved. However, under extreme situations such as sudden load changes or strongly nonlinear effects, the predefined QFT templates may be exceeded. This represents a general limitation of robust control methods, as no adaptation is included, and it is not a deficiency of the chosen method or an error in the controller design. In such cases, further extension of the templates or the use of more complex Multiple Input, Multiple Output (MIMO) models would be required, which is beyond the scope of the present study.

### 4.5. Modeling of the Individual Axes

#### 4.5.1. Modeling Assumptions and Axis Dynamics

After decoupling the dynamic model of the robot, each joint $J_{i}$ can be treated as an independent SISO plant whose dynamics are dominated by the drive motor and the mechanical inertia of the corresponding link, as shown in Figure 9. For practical control purposes, the plant is approximated by the dynamic model given in (23).

The model (23) is valid within the operating workspace of the manipulator, where nonlinear effects such as friction, backlash, and gravity are treated as external disturbances (27), while the cross-axis interactions are captured through the parametric variations of $k_{i}$ and $T_{i}$.

Under this formulation, the regulators $C_{i}$ for each of the joints $J_{i}$ ensure accurate tracking of the reference angle $\theta_{\mathrm{ref},i}(t)$. The goal is to drive the tracking error (29) to zero through the control input $u_{i}$ applied to the plant, such that the actual joint motion $\theta_{i}(t)$ follows the desired trajectory $\theta_{\mathrm{ref},i}(t)$.

(29) $$e_{i}\left(t\right)=\theta_{ref,i}\left(t\right)-\theta_{i}\left(t\right)=\underset{t\to \infty }{\lim}\;e_{i}\left(t\right)=0.$$

The values in Table 5 represent the nominal parameters of the individual axes $J_{i}$ and their corresponding variations, which are used for generating the QFT templates and for analyzing the worst-case dynamics.

The parameters $k_{i}$ and $T_{i}$ in Table 5 describe the control-oriented dynamic characteristics of the five actuated axes $J_{i}$ of the anthropomorphic manipulator FANUC M-430iA/4FH, represented as a dynamic plant with transfer Function (23). Specifically, the parameters $k_{1},T_{1}$ characterize the base rotation (axis $J_{1}$), where the overall structural inertia dominates. The parameters $k_{2},T_{2}$ correspond to the shoulder motion (axis $J_{2}$), which is the most inertial and slowest axis due to the large gravitational moment. The parameters $k_{3},T_{3}$ describe the elbow joint (axis $J_{3}$), exhibiting medium dynamics and moderate parameter variations. The parameters $k_{4},T_{4}$ and $k_{5},T_{5}$ characterize the two wrist axes (axes $J_{4}$ and $J_{5}$), which are defined by low inertia and the highest response speed.

The parameter ranges $\left(k_{i,min},k_{i,max}),(T_{i,min},T_{i,max}\right)$ in Table 5 are selected to cover the expected variations arising from different robot configurations, payload changes, and dynamic couplings between the axes. These ranges are used to construct the QFT templates and to identify the most adverse parameter combinations (worst-case dynamics), which define the boundary conditions for synthesizing the controllers $C_{i}(s)$.

The dynamic parameters of the axis models presented in Table 5 were determined based on the nominal dynamic model of each axis and engineering estimates of the allowable variations in gain and time constants under different loading conditions. They reflect changes resulting from variations in payload mass, mechanical inertia, and the operating configuration of the FANUC M-430iA/4FH manipulator, within the manufacturer’s specifications.

#### 4.5.2. Modeling of Disturbance Influence

In addition to the nominal dynamics of each axis $J_{i}$, the real robotic system is affected by various internal and external disturbances that influence the joint position $\theta_{i}$. The primary disturbance sources typically include variable payload forces exerted by the manipulated object or tool, friction in the joints and gear reducers, mechanical backlash, and dynamic couplings between adjacent axes. To account for their influence on the controlled coordinate $\theta_{i}(t)$, the system is augmented with an additional disturbance channel that represents the path of the disturbance $G_{\zeta_{M}}(s)$ to the output $\theta_{i}(s)$, as illustrated in Figure 9.

Empirically, this disturbance channel is modeled as a linear transfer function corresponding to an external torque- or force-induced disturbance, given by the following (30):

(30) $$G_{\zeta_{M}}\left(s\right)=-\frac{0.253\left(1.5s+1\right)}{s\left(0.01s+1\right)\left(6s+1\right)}.$$

The denominator represents the inertial and damping characteristics of the mechanical structure, while the numerator includes the dynamic zero term (1.5s+1), associated with the elastic coupling between the tool and the manipulator. The negative sign reflects the fact that an increase in the external disturbance force produces a deviation in the direction opposite to the commanded trajectory.

The transfer function describing the external-disturbance dynamics in (30) was identified through simulation experiments in ROBOGUIDE, where a moment disturbance $\zeta_{M}(t)$ was applied to the digital twin and the corresponding response in the joint coordinate $\theta_{i}(t)$ was analyzed in MATLAB.

The resulting third-order transfer Function (30) was selected as a compromise between model adequacy and structural simplicity. In the context of QFT analysis, this channel is treated as an output disturbance acting on each plant (23) and is used to evaluate the disturbance sensitivity, from which the disturbance-rejection bounds are derived. The overall objective of the QFT controller is to minimize the influence of $G_{\zeta_{M}}(s)$ on the output $\theta_{i}(s)$ at low frequencies while maintaining stability and satisfactory dynamic performance within the operational bandwidth.

In Section 4.7, model (30) is used in forming the QFT bounds for all five controllers $C_{i}$, since the moment the disturbance propagates through the entire kinematic chain. The strongest effect is observed at the terminal axis $J_{5}$ due to the cumulative structural dynamics along the manipulator arm, as illustrated in Figure 9.

#### 4.5.3. Modeling of Parametric Variations and Worst-Case Dynamics

In a real robotic system, the dynamics of each joint $J_{i}$ vary as a function of the joint position $\theta_{i}$, the applied load, and the interactions between the links. To account for this uncertainty, the plant parameters $k_{i}$ and $T_{i}$ are varied within the ranges presented in Table 5. The nominal model of each axis is described by the transfer function (31):

(31) $$G_{i,nom}(s)=\frac{k_{i,nom}}{s(T_{i,nom}s+1)},\;i=1,\ldots ,5$$

which serves as the reference point for QFT controller design in the Nichols chart.

The actual dynamics under different parameter combinations are represented by the model (32):

(32) $$G_{i}\left(s\right)=\frac{k_{i}}{s\left(T_{i}s+1\right)}=G_{i,nom}\left(s\right)\;\left[1+w_{i}\left(s\right)\right].$$

For controller synthesis, the worst-case dynamics are determined, that is, the parameter combination corresponding to the largest values of $k_{i}$ and $T_{i}$, given by the following (33):

(33) $$G_{i,worst}\left(s\right)=\frac{k_{i,max}}{s\left(T_{i,max}s+1\right)},\;i=1,\ldots ,5.$$

Within the QFT methodology, the templates define the regions of uncertainty in which the controllers $C_{i}$ must ensure stability and performance for all admissible values of the parameters $k_{i}$ and $T_{i}$. In this way, accurate tracking of the joint positions of the five axes $J_{i}$, represented by the angular coordinates $\theta_{i}(s)$, is guaranteed across the entire operating envelope.

### 4.6. QFT Algorithm Overview [8,9,10]

The synthesis procedure of a QFT controller for each joint $J_{i}$ consists of several fundamental steps:

- Plant modeling. The nominal (31) and perturbed (32) models from Table 5 are selected. These models are used to construct the QFT plant templates in the Nichols plane.
- Template construction. For selected frequencies, referred to as essential frequencies $\omega_{k}$, the magnitude-phase characteristics of all admissible variants of the plant dynamics $G_{i}(j\omega_{k})$ are computed. The resulting envelopes form the plant templates, which represent parametric uncertainty.
- Bounds specification. Robust performance bounds are defined: tracking bounds $B_{\mathrm{tr}}(\omega_{k})$, ensuring low sensitivity at low frequencies; disturbance-rejection bounds $B_{D}(\omega_{k})$, derived from the disturbance model $G_{\zeta_{M}}(s)$; a universal high-frequency bound (the U-contour), which specifies a region that the frequency response of the designed open-loop system must not enter. Together, these constraints define the optimal bounds $B_{O}(\omega_{k})$, which the QFT controller must satisfy.
- Loop shaping. In the Nichols plane, the frequency response of the optimal open-loop system $L_{i}(j\omega )=G_{i,\mathrm{nom}}(j\omega )C_{i}(j\omega )$ is shaped so that it satisfies all templates and bounds based on the nominal plant model and the optimal limits $B_{O}(\omega_{k})$.
- Prefilter design. The prefilter $F_{i}(s)$ is used to refine the transient response and limit its aggressiveness. Its purpose is to shape the closed-loop frequency response so that the resulting time-domain behaviors, specifically the joint trajectories $\theta_{i}(t)$ and their settling characteristics, meet the required performance criteria.
- Verification. Closed-loop stability and performance are verified for all admissible plant variations $G_{i}(s)$ and under the influence of the disturbance channel $G_{\zeta_{M}}(s)$.

The dynamics of each of the five axes of the anthropomorphic manipulator are represented by the reduced first-order model with an integrating term (23). For every joint, a parametric uncertainty region is defined as (34), where the nominal and extreme (worst-case) combinations of the parameters $\Omega_{i}$ are obtained according to Table 5.

(34) $$\Omega_{i}=\{\;(k,T_{i})\;|\;k_{i}\in [k_{i,min},k_{i,max}],\;T_{i}\in [T_{i,min},T_{i,max}]\;\}$$

The parametric uncertainty for each axis can be expressed through the nominal and worst-case plant models. The five joints exhibit distinct electromechanical characteristics:
$J_{1}$Base rotation:$G_{1,\mathrm{nom}}(s)=\frac{3.85}{s\left(1.91s+1\right)}$$G_{1,\mathrm{worst}}(s)=\frac{8.58}{s\left(5.0s+1\right)}$$J_{2}$Shoulder motion:$G_{2,\mathrm{nom}}\left(s\right)=\frac{0.25}{s\left(6.0s+1\right)}$$G_{2,\mathrm{worst}}(s)=\frac{0.753}{s(11s+1)}$$J_{3}$Elbow joint:$G_{3,\mathrm{nom}}(s)=\frac{3.85}{s(1.5s+1)}$$G_{3,\mathrm{worst}}(s)=\frac{3.85}{s(4.5s+1)}$$J_{4}$Wrist rotation (yaw):$G_{4,\mathrm{nom}}(s)=\frac{2.5}{s(1.2s+1)}$$G_{4,\mathrm{worst}}(s)=\frac{3.0}{s(2.0s+1)}$$J_{5}$Wrist rotation (pitch/roll):$G_{5,\mathrm{nom}}(s)=\frac{2.0}{s(1.0s+1)}$$G_{5,\mathrm{worst}}(s)=\frac{2.8}{s(1.8s+1)}$

Thus, the parametric uncertainty for each axis can be represented as a compact set $P_{i}$ (35):

(35) $$P_{i}=\{G_{i}(s)\;|\;(k_{i},T_{i})\in \Omega_{i}\},$$

where the transfer function (31) represents the nominal point of the set $P_{i}$, corresponding to the combination of the minimum parameter values $k_{i}$ and $T_{i}$. The QFT plant templates are constructed for a vector of essential frequencies $\omega_{k}$ (36), which best capture the dynamics of the corresponding axis $J_{i}$; that is, these frequencies span approximately two decades around the break frequency $\omega_{b}$ of the nominal plant (31).

(36) $$\omega_{k}\in \left[0.1\;\omega_{b},\;10\;\omega_{b}\right],\;k=1\ldots 5$$

Accordingly, the following vectors $\omega_{k}$ of essential frequencies are obtained for the axes $J_{1}$ through $J_{5}$, as given in (37).

(37) $$\begin{array}{c} J1:\;\;\omega_{1}\in \left[0.05,0.1,0.5,1,3,5,10\right]\\ J2:\;\;\omega_{2}\in \left[0.01,0.05,0.1,0.5,1,5\right]\\ \;\;J3:\;\;\omega_{3}\in \left[0.01,0.05,0.1,0.5,1,5,10\right]\\ \;\;J4:\;\;\omega_{4}\in \left[0.01,0.05,0.1,0.5,1,5,10\right]\\ J5:\;\;\omega_{5}\in \left[0.05,0.1,0.5,1,5,10\right] \end{array}$$

**Figure 10:**
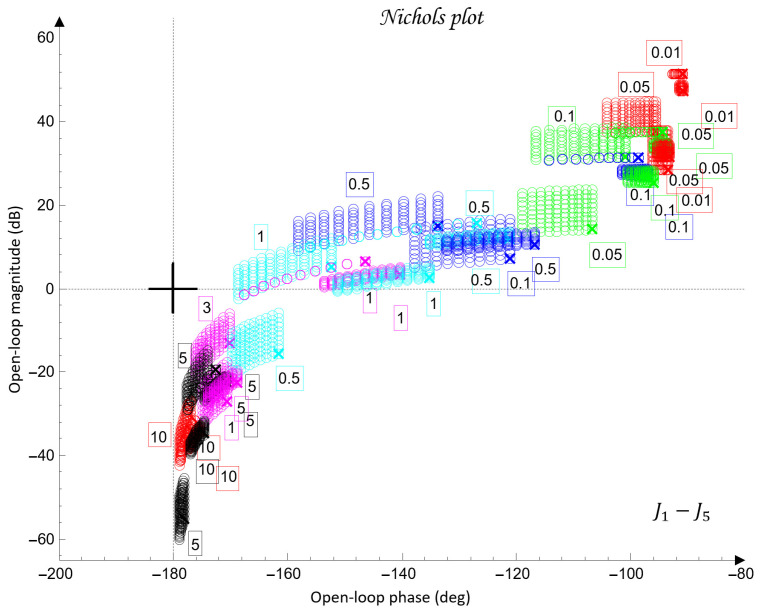
QFT plant templates in the Nichols chart for axes $J_{1}–J_{5}$ under parametric uncertainty.

Figure 10 shows the QFT plant templates of the dynamic models of the robot axes in the frequency domain on the Nichols plane.

The uncertainty regions obtained in Figure 10 clearly illustrate the pronounced variations in the dynamics of the joints $J_{i}$, caused by load changes, friction effects, and cross-axis interactions. The wide spread of the plant templates confirms the presence of significant uncertainty in the model (23), making the use of robust QFT synthesis essential for ensuring stable and high-quality tracking of the joint coordinates $\theta_{i}$ under all admissible operating conditions.

Before constructing the robust bounds, it is necessary to specify the admissible performance criteria PC (38) and (39) for each joint $J_{i}$, consistent with the physical constraints of the robot and its DH parameters. These criteria are formulated as prototype models of the desired closed-loop behavior and define lower and upper limits on the output variable $\theta_{i}$, in accordance with the QFT methodology.

(38) $$\begin{array}{c} J1\;\mathrm{PC}:\;\left\{t_{s}\left(2\%\right)\leq 2.5\;s,\;\sigma \leq 10\%\right\};\\ J2\;\mathrm{PC}:\;\left\{t_{s}(2\%)\leq 6.0s,\;\sigma \leq 8\%\right\};\;\\ J3\;\mathrm{PC}:\;\left\{t_{s}(2\%)\leq 2.0\;s,\;\sigma \leq 10\%\right\};\\ J4\;\mathrm{PC}:\;\left\{t_{s}\left(2\%\right)\leq 1.2s,\;\sigma \leq 12\%\right\};\\ J5\;\mathrm{PC}:\;\left\{t_{s}(2\%)\leq 1.0s,\;\sigma \leq 15\%\right\};\\ \mathrm{Position}\;\mathrm{step}\;\mathrm{error}:\;|e_{ss}|\;=0. \end{array}$$

Trajectory (spline): The reference 3D trajectory is defined through five control points, as given in (19).

$$\begin{array}{c} P_{1}=\left(0.40,\;0.10,\;0.30\right),\\ P_{2}=\left(0.45,\;0.13,\;0.33\right),\;\\ P_{3}=\left(0.50,\;0.17,\;0.345\right),\\ P_{4}=\left(0.55,\;0.21,\;0.355\right),\\ P_{5}=(0.60,\;0.25,0.360),\; \end{array}$$

where the smooth desired trajectory is generated using a cubic spline over the normalized parameter s(t).

The performance criterion for QFT takes the following form (39):

(39) $$PC:\underset{t\in [0,25]}{max}∥p_{ref}(t)-p_{TCP}(t)∥\;\leq 1\;\mathrm{mm},$$

where $p_{ref}(t)$ is the desired B-spline trajectory and $p_{TCP}(t)$ is the resulting end-effector position. It should be noted that the criterion in (39) reflects the industrial requirement applied to the real FANUC controller, which employs gravity compensation, feedforward structures, and Look Ahead planning. These mechanisms allow the real system to achieve sub-millimeter accuracy in the TCP space.

In the simulation model, which uses the simplified first-order SISO dynamics (23) without nonlinear compensation and without the internal high-frequency FANUC algorithms, larger deviations from the geometric spline trajectory are expected. Therefore, for the purpose of model-based analysis, it is appropriate to introduce an additional operational criterion based on the maximum 3D error observed under nominal and worst-case dynamic models (40):

(40) $$PC_{sim}:\underset{t\in [0,25]}{max}∥p_{ref}(t)-p_{TCP}(t)∥\;\leq 20\;\mathrm{mm},$$

which reflects the dynamic limitations of the simplified model without contradicting the stricter industrial requirement given by (39).

Thus, the two criteria serve different purposes: (39) corresponds to the real engineering specification for the physical robot, whereas the operational criterion (40) provides the basis for evaluating robustness in the simulation environment. Criterion (40) ensures that the end effector follows the prescribed spline within an admissible error bound suitable for both simulation and industrial applicability.

Additionally, for the purposes of simulation-based robustness assessment, quantitative error metrics are introduced in both the joint space and the TCP space, defined through the maximum error and the RMS value. Let the total 3D error between the desired and the realized TCP trajectory be denoted by the following (41):

(41) $$e_{3D}(t)=\;∥p_{ref}(t)-p_{TCP}(t)∥,$$

where the maximum and RMSs are defined as (42)

(42) $$e_{3D,max}=\underset{t\in \left[0,T\right]}{\max e_{3D}\left(t\right)},e_{3D,RMS}=\sqrt{\frac{1}{T}\int_{0}^{T}e_{3D}^{2}\left(t\right)\;dt}.$$

These metrics are later used in Section 6 to validate the control performance and to compare the nominal and worst-case joint models.

The following pairs of desired closed-loop systems (43) are defined, specifying the target joint-space dynamics for the robot joints $J_{i}$, whose dynamic parameters satisfy the constraints in (38).

(43) $$\begin{array}{c} J1:\;T_{U}=\frac{12.25}{s^{2}+4.9s+12.25}\;T_{L}=\frac{12.25}{s^{2}+4.9s+12.25}\\ J2:\;T_{U}=\frac{1}{s^{2}+2s+1}\;T_{L}=\frac{1}{1.5s+1}\\ J3:\;T_{U}=\frac{16}{s^{2}+4s+16}\;T_{L}=\frac{27}{s^{3}+9s^{2}+27s+27}\\ J4:\;T_{U}=\frac{1}{0.3s+1}\;T_{L}=\frac{64}{s^{3}+12s^{2}+48s+64}\\ J5:\;T_{U}=\frac{1}{s^{2}+8s+64}\;T_{L}=\frac{125}{s^{3}+15s^{2}+75s+125} \end{array}$$

During the design of (43), it is taken into account that the difference in the magnitudes $\delta_{tr}(\omega_{k})$ between the closed-loop systems $T_{U}$ and $T_{L}$ in the frequency domain must continuously increase in order to cover, during the QFT controller synthesis, the high-frequency uncertainty (44).

(44) $$\delta_{tr}\left(\omega_{k}\right)=\left|T_{U}\left(j\omega_{k}\right)\right|-\left|T_{L}\left(j\omega_{k}\right)\right|.$$

**Figure 11:**
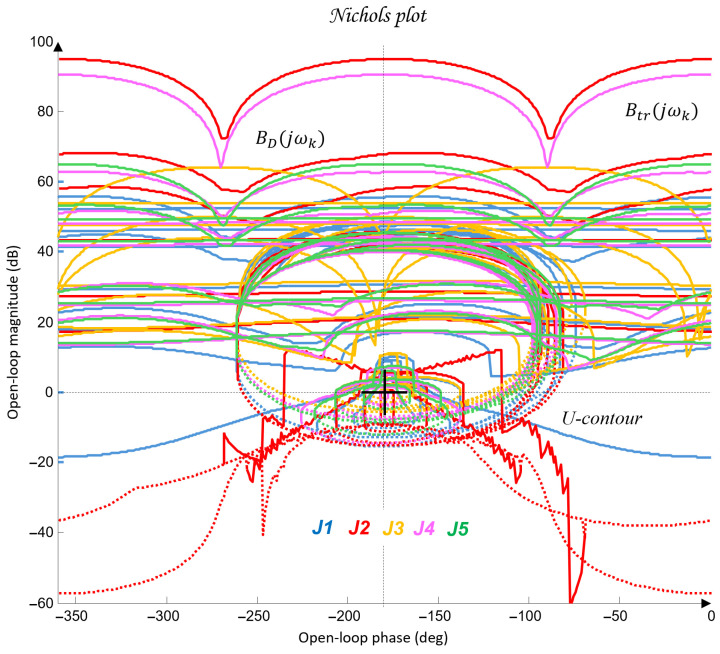
Nichols plot of QFT bounds showing $B_{tr}(j\omega_{k})$, $B_{D}(j\omega_{k})$, U-contour for robot joints $J_{1}–J_{5}$.

Next, three robust bounds are constructed: the tracking bounds $B_{tr}(j\omega_{k})$, defined by (38); the output-disturbance bound $B_{D}(j\omega_{k})$, derived from (30); and the formation of the U-contour, which specifies a forbidden region for the open-loop frequency response in the Nichols plane around the critical point $\left(-180^{o},0\;dB\right)$. The magnitude of the U-contour is determined by the weighting factor $w_{\text{U-contour}}=1.01,$ which ensures that the transient responses of the joint coordinates $\theta_{i}$ exhibit no overshoot. All robust bounds are plotted in the frequency domain for the vectors of essential frequencies (37) for each of the five joints $J_{i}$, using the uncertainty regions of the five plants $G_{i}$ (35) and comparing their position relative to the iso-magnitude curves in the Nichols plane and the difference $\delta_{tr}(\omega_{i})$, following the standard QFT procedure. Figure 11 shows the robust bounds for each joint of the robot: $J_{1}$ in blue, $J_{2}$ in red, $J_{3}$ in yellow/orange, $J_{4}$ in purple, and $J_{5}$ in green, corresponding to the vector of essential frequencies $\omega_{k}$ (37).

**Figure 12:**
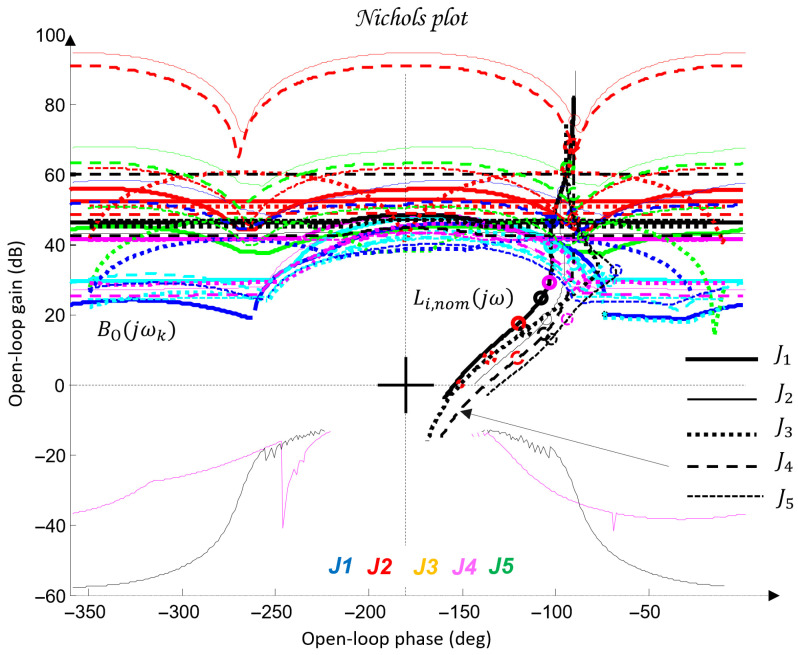
QFT loop-shaping results in the Nichols domain: nominal open-loop responses $L_{i,nom}\left(j\omega \right)$ with controllers $C_{i}\left(j\omega \right)$ relative to the bound $B_{O}(j\omega_{k})$ for joints $J_{1}–J_{5}$.

The final step before designing the joint QFT controllers $C_{i}$ consists of optimizing the bounds from the network shown in Figure 11. This is performed by comparing, at each essential frequency, the tracking bounds $B_{tr}(j\omega_{k})$, the disturbance-rejection bounds $B_{D}(j\omega_{k})$, and the U-contour. For every frequency point, the uppermost bound is selected. In this way, the optimal bounds $B_{O}(j\omega_{k})$ are constructed, ensuring that the controllers are synthesized for the worst-case combination of the plant parameters of the dynamic models (23), as illustrated in Figure 12.

### 4.7. Controller Synthesis and Validation

Loop shaping is carried out by designing a QFT controller that ensures low closed-loop sensitivity and a sufficiently large closed-loop bandwidth. The resulting transfer function of the QFT controller $C_{i}(s)$, obtained following the QFT methodology, is of low order, since the regulator design is based on the nominal joint models of the robot (35) and on the optimal bounds $B_{O}(j\omega_{k})$.

The outcome of this design step is the set of optimal open-loop frequency responses $L_{i,nom}(j\omega )$, which lie on or above the corresponding robust optimal bounds $B_{O}(j\omega_{k})$ for all essential frequencies $\omega_{k}$(37), as illustrated in Figure 12.

For the selection of the controllers $C_{i}$, loop shaping is performed by augmenting the complex transfer function of the nominal open-loop system (45) with poles and zeros until an optimal solution is obtained (46):

(45) $$L_{i,nom}\left(j\omega \right)=G_{i,nom}\left(j\omega \right)$$

(46) $$L_{i,nom}\left(j\omega \right)=C_{i}\left(j\omega \right)G_{i,nom}\left(j\omega \right).$$

The transfer functions of the QFT controllers $C_{i}(s)$ for the individual joints $J_{i}$ are given by the following (47):

(47) $$\begin{array}{c} J1:\;C_{1}\left(s\right)=\frac{42.52s\;+\;31.95}{0.05307s\;+\;1}\\ J2:\;C_{2}\left(s\right)=\frac{10.66s\;+\;23.74}{0.05528s\;+\;1}\\ J3:\;C_{3}\left(s\right)=\frac{13.67s\;+\;4.3}{0.1014s\;+\;1}\\ J4:\;C_{4}\left(s\right)=\frac{12.84s\;+\;10.27}{\;0.05869s\;+\;1}\\ J5:\;C_{5}\left(s\right)=\frac{22.68\;s\;+\;7.43}{\;0.01966s\;+\;1} \end{array}$$

Figure 12 presents the optimal open-loop frequency responses of the five systems with the designed QFT controllers (47) and the nominal plant (35). The frequency characteristics in Figure 12 show that the closed-loop bandwidths of the joint controllers $J_{i}$ are wide, since the added lead dynamics of the controllers $C_{i}$ shift the open-loop response (46) to the right.

**Figure 13:**
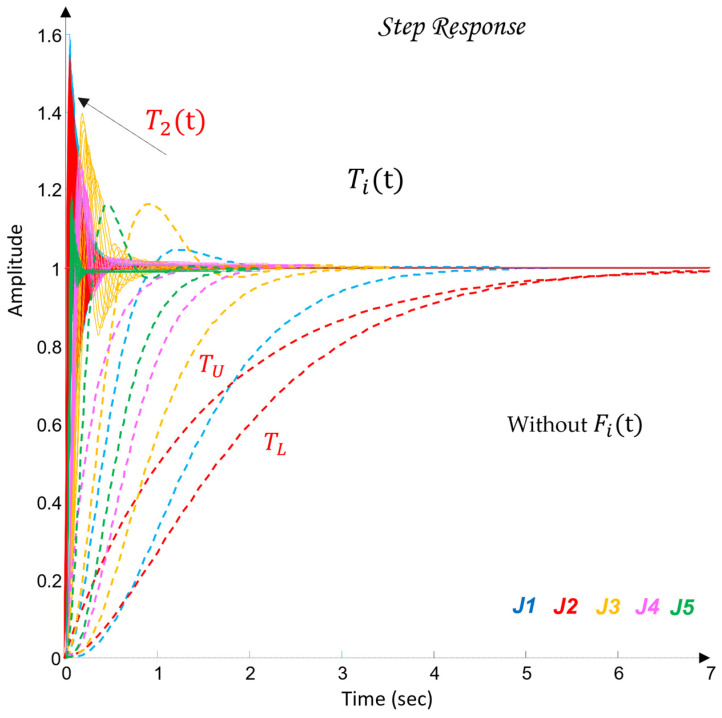
Closed-loop step responses $T_{i}(t)$ obtained with QFT controllers only (without the prefilter $F_{i}$), illustrating the tracking performance bounds $T_{L}$, $T_{U}$, and $T_{2}(t)$ (shown for $J_{2}$ in red) for joints $J_{1}–J_{5}$.

Figure 13 shows the time-domain transient responses of the closed-loop systems $T_{i}$ for the individual joints $J_{i}$, plotted in different colors.

The closed-loop responses $T_{i}$ shown in Figure 13 are obtained in a simulation environment under a unit step input. It is evident that the responses are very fast and do not guarantee compliance with the performance requirements (38), defined by the desired envelopes $T_{U}$ and $T_{L}$. This discrepancy highlights the role of the designed QFT controllers $C_{i}$ (47).

The final step of the QFT methodology consists of designing an additional dynamic element applied at the input of the joint-level control system of each axis $J_{i}$. Its purpose is to ensure the desired control performance of the joints through proper shaping of the reference position $\theta_{i}$. This task is accomplished by synthesizing a prefilter $F_{i}$ (48), whose dynamics slow down and shape the response of the existing QFT-controlled system, thereby ensuring robustness and the required performance of the closed-loop system $T_{PC}^{F}(s)$, as illustrated in Figure 9.

**Figure 14:**
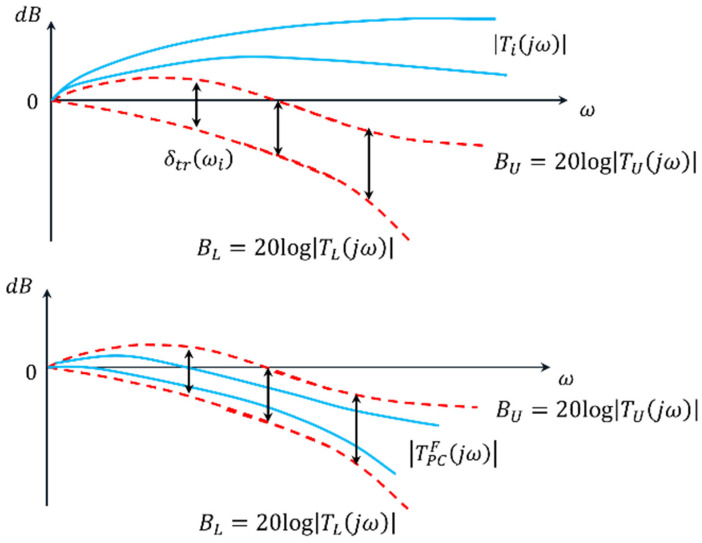
Frequency-domain synthesis of prefilters (|F||T| alignment).

The synthesis of the prefilter $F_{i}$ is performed in the frequency domain for each of the five joints $J_{i}$. In the present case, two poles are added to shift the magnitude of the closed-loop frequency response of the QFT-controlled system by approximately −40 dB, followed by fine-tuning of the pole locations, as shown in Figure 14.

(48) $$\left|F_{i}\left(j\omega \right)\right|\left|T_{i}\left(j\omega \right)\right|=\left|{T_{i}}_{PC}^{F}\left(j\omega \right)\right|$$

The following prefilter transfer functions $F_{i}(s)$ have been designed as part of the joint-level control system for the axes $J_{i}$ (49):

(49) $$\begin{array}{c} J1:\;F_{1}\left(s\right)=\frac{1}{0.146s^{2}+0.9253s+1}\;\\ J2:\;F_{2}\left(s\right)=\frac{1}{0.1796s^{2}\;+\;1.65s\;+\;1}\;\\ J3:\;F_{3}\left(s\right)=\frac{1}{0.09552s^{2}+\;0.6925s\;+\;1}\\ J4:\;F_{4}\left(s\right)=\frac{1}{0.0313s^{2}\;+\;0.4798s\;+\;1}\\ J5:\;F_{5}\left(s\right)=\frac{1}{0.0313s^{2}\;+\;0.3553s\;+\;1} \end{array}$$

**Figure 15:**
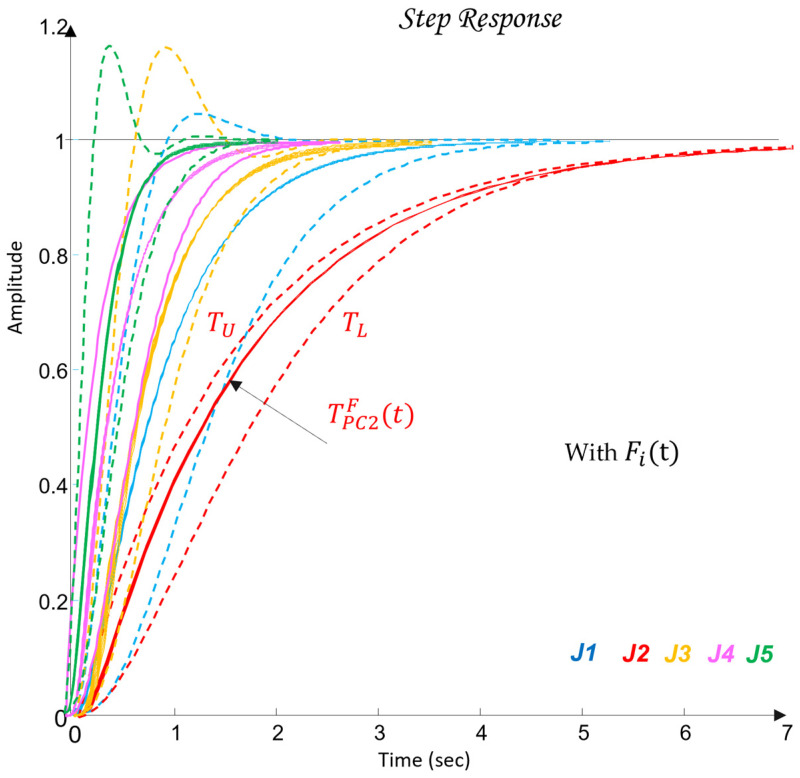
Closed-loop step responses $T_{PC}^{F}(t)$ obtained with QFT controllers and prefilters $F_{i}$, illustrating the tracking performance bounds $T_{L}$ and $T_{U}$; the filtered response $T_{PC}^{F}(t)$ for joint $J_{2}$ is shown in red (joints $J_{1}–J_{5}$).

Figure 15 shows the transient responses of the closed-loop control systems $T_{PC}^{F}$ for the robot joints $J_{i}$.

It is evident that the dynamics of the prefilters $F_{i}$ ensure the desired control quality specified in (38), as well as robustness of the closed-loop system, since the model uncertainty in the joint dynamics $J_{i}$ (Figure 10) is practically eliminated. In other words, the entire set of plant models between $G_{i,nom}(s)$ and $G_{i,worst}(s)$, whose behavior is regulated by $C_{i}$ and $F_{i}$, results in a family of transient responses that visually collapse into a single trajectory.

The design of the QFT controllers and prefilters follows the Quantitative Feedback Theory controller and prefilter design procedure described in [8,9,10]. For each of the five axes, the controller is designed by frequency shaping of the nominal open-loop system (45) and (46), assuming a nominal plant model and an initial proportional controller with a gain equal to 1. The open-loop dynamics are modified by adding poles and zeros to the transfer function (46). The objective is for the open-loop system to satisfy the optimal frequency-domain bounds defined by the uncertainty templates and the specified requirements for robust tracking and disturbance rejection (Figure 12). The obtained solution represents one possible optimal solution within the QFT procedure; other transfer functions may also satisfy the same bounds and requirements (47), but their investigation is beyond the scope of the present study. The purpose of the QFT controllers is to increase the closed-loop bandwidth (Figure 13), while the prefilter dynamics are used to slow down the system response until the performance criterion is satisfied.

The prefilter transfer function is obtained by adding poles and zeros to the closed-loop transfer function with the QFT controller (48) until the performance criterion is met and the resulting frequency characteristics fall within the desired performance region (Figure 14). The resulting prefilter transfer function is optimal within the adopted constraints (49), but it is not unique and may be the subject of further investigation (Figure 15).

### 4.8. Discrete-Time Realization of the QFT Controllers and Prefilters

Although the synthesis of the QFT controllers $C_{i}(s)$ and the prefilters $F_{i}(s)$ in this work is performed in the continuous time domain, their actual implementation within the PLC-MATLAB-digital twin control loop is carried out in discrete time. The discretization is aligned with the architecture presented in Section 3, where the control cycle operates with a fixed sampling period of $T_{s}=20\;\mathrm{ms},$ corresponding to a sampling frequency of 50 Hz. This frequency is approximately 15–20 times higher than the crossover frequency of the optimal open-loop systems obtained during the QFT loop-shaping procedure in Section 4.6. As a result, the discrete-time realization of the five joint subsystems preserves the essential phase characteristics and does not violate the robustness constraints imposed by the templates and the bounds $B_{tr}(\omega_{k})$, $B_{D}(\omega_{k})$, and $B_{O}(\omega_{k})$. The chosen discretization approach follows the conclusions of earlier studies in [56] on frequency-oriented QFT synthesis, where the Tustin method (bilinear transformation) consistently demonstrated the best compatibility between analog and digital closed-loop behavior in the presence of parametric uncertainty. The Tustin method preserves the local shape of the frequency response around the crossover region and minimizes the uncertainty introduced by discretization—an essential requirement for maintaining the correct behavior of the U-contour. In contrast, Zero-Order Hold (ZOH) discretization introduces significant phase distortion around the Nichols-plot crossing region, which may lead to violations of robustness constraints and changes in relative stability. For this reason, ZOH is not suitable in a QFT context. The 20 ms sampling period is also consistent with the PLC layer, which supports an event-driven control tick of 20 ms, independent of its internal scan cycle. This ensures conflict-free integration between the continuous-time model, the digital realization in MATLAB, and the actual execution within the PLC–ROBOGUIDE environment, without introducing additional latency or undesirable phase shifts. Consequently, the discretized transfer functions of the controllers $C_{i}(z)$ (50) and the prefilters $F_{i}\left(z\right)$ (51) operate correctly and in real time within the MATLAB–PLC–ROBOGUIDE loop, where the PLC provides deterministic communication through OPC UA and Modbus TCP, in accordance with the architecture described in Section 3.

(50) $$\begin{array}{c} C_{1}(z)=\frac{679.2z-669.1}{z-0.6829}\\ C_{2}(z)=\frac{163.7z-162.9}{z-0.6936}\\ C_{3}(z)=\frac{123.1z-122.3}{z-0.8205}\\ C_{4}\left(z\right)=\frac{188.4z-185.4}{z-0.7088}\\ C_{5}(z)=\frac{767.2z-762.2}{z-0.3257} \end{array}$$

(51) $$\begin{array}{c} F_{1}(z)=\frac{0.0006437z^{2}+0.001287z+0.0006437}{z^{2}-1.878z+0.8809}\\ F_{2}(z)=\frac{0.0005097z^{2}+0.001019z+0.0005097}{z^{2}-1.83z+0.8318}\\ F_{3}(z)=\frac{0.0009752z^{2}+0.00195z+0.0009752}{z^{2}-1.861z+0.8649}\\ F_{4}(z)=\frac{0.002763z^{2}+0.005525z+0.002763}{z^{2}-1.724z+0.7349}\\ F_{5}(z)=\frac{0.002861z^{2}+0.005722z+0.002861}{z^{2}-1.785z+0.7967} \end{array}$$

The resulting discrete-time transfer functions (50) and (51) are used directly in real time within the MATLAB-PLC-ROBOGUIDE control loop (Section 6), where they are validated against both nominal and worst-case models. In this way, the discretization ensures correct digital execution of the QFT control law, preserves stability and accuracy under worst-case dynamics and external disturbances, and guarantees full compatibility with the communication constraints of the CPDTQN architecture, as demonstrated in the results presented in Section 6.3 and Section 6.4.

## 5. Digital Twin Integration and NIS2 Security Compliance

In the context of the expanding digitalization associated with Industry 4.0 and Industry 5.0, industrial robotic systems have evolved from isolated mechanical devices into fully integrated cyber-physical environments in which PLCs, robotic manipulators, cloud services, digital twins, and intelligent control algorithms exchange data in real time [53,54,55]. This integration significantly increases the adaptability and efficiency of manufacturing processes, while simultaneously expanding the system’s attack surface [54]. Communication protocols, telemetry streams, digital twin models, and configuration resources become critical points exposed to risks such as unauthorized access, data manipulation, software compromise, or injection of malicious behavioral models [22,23,42].

Against this background, Directive (EU) 2022/2555 (NIS2) introduced an expanded pan-European regulatory framework with requirements for risk management, cyber-resilience, incident reporting, supply-chain protection, and continuity of critical services [22,23,40]. Although industrial robots are not defined as independent sectors, they fall within regulated categories related to the production of critical goods, digital infrastructure, and ICS/SCADA (Industrial Control System/Supervisory Control and Data Acquisition) environments [22,40]. Consequently, the integration of digital twins and robust control must be assessed not only from a technical perspective but also in terms of regulatory requirements for security, traceability, and accountability.

Within the CPDTQN architecture (Figure 5), the digital twin operates as a synchronized model of the physical robot, maintaining continuous bidirectional exchange of states, parameters, and control variables [53,54]. This enables virtual diagnostics, simulation-based validation, monitoring, and disturbance response, while simultaneously requiring secure data exchange among the MATLAB/QFT controller, the PLC, and the twin environment. Quantitative Feedback Theory provides the formal framework needed for synthesizing robust control laws under parametric uncertainty and external disturbances [42,54], and in this work it is combined with NIS2-oriented mechanisms for access control, cryptographic protection, logging, and risk management. The resulting multilayer configuration ensures resilience at three levels: technical resilience through the robust controller, operational resilience through monitoring and simulation in the digital twin, and regulatory resilience through compliance with the NIS2 requirements. The specific alignment between the architecture and the regulatory framework is summarized in Table A1, where the implemented measures are mapped to the corresponding articles of the directive.

### 5.1. PLC Communication with the Digital Twin (Modbus TCP/IP)

The communication between the PLC and the digital twin is implemented via Modbus TCP/IP with additional TLS encryption, where the PLC operates as the master device and the digital twin as the slave device [54]. This channel is used to transmit the control inputs $u_{i}(t)$, the measurements $\theta_{i}(t)$ for the five axes, as well as the accompanying telemetry—positions, velocities, currents, and input/output module states. The same mechanism is also used for transmitting alarms, diagnostic states, and synchronization data between the physical system and the virtual model, ensuring real-time bidirectional exchange [54].

Records for positions, loads, variable friction, velocity profiles, and diagnostic flags are logged and synchronized using UTC timestamps, which enables continuous monitoring and full event recoverability. This approach provides traceability and accountability, required for ensuring compliance with NIS2 obligations related to access control, logging, and risk management.

The Modbus TCP/IP protocol, although widely adopted in industrial practice, does not inherently include mechanisms for encryption, authentication, or integrity verification [54]. As a result, it is susceptible to eavesdropping, command tampering, MITM attacks, replay scenarios, falsified measurements, noise injection, and firmware compromise in the absence of secure update mechanisms. In the context of NIS2, these risks are particularly significant for robotic systems classified as critical manufacturing components [22,23]. In the present implementation, cryptographic protection is provided through TLS encapsulation (VPN tunneling or protocol wrapping), which adds confidentiality, integrity, and authenticity to Modbus TCP/IP communications, even though the protocol itself does not provide such mechanisms by design.

The implemented CPDTQN architecture mitigates these vulnerabilities through role-based access control and authentication executed at the PLC level, which acts as a secure gateway between the control algorithm and the actuation mechanisms [22,23]. Additional protection is provided through Modbus TCP with TLS or via OPC UA with enabled Security Policies [54]. All critical events and command sequences are logged in parallel within both the PLC and MATLAB, enabling reconstruction of the complete interaction timeline and facilitating compliance verification and forensic analysis [9].

The digital twin environment functions both as a state redundancy layer and as a simulation-based recovery and risk assessment tool, allowing test scenarios involving parameter variations, communication delays, or controlled cyber incidents [42,53,54].

In this form, the communication scheme extends the classical closed-loop structure by adding a layer of observability, reliability, and cyber-resilience, with the implemented mechanisms following the Protect–Detect–Respond–Recover principles embedded in the NIS2 directive.

### 5.2. Simulated Disturbances and Loads in the Digital Twin

The digital twin environment enables controlled reproduction of a wide spectrum of dynamic scenarios through which the behavior of the QFT controller is validated under parametric uncertainty, disturbances, and cyber incidents. The virtual environment supports the application of physical effects such as variations in inertial parameters, changes in friction, external forces, impacts, and asymmetric loading of the manipulator. In addition to these physical influences, sensor-related anomalies are also modeled, including noise, drift, intermittent faults, or partial loss of measurement signals. A third category of scenarios includes communication disturbances manifested through delays, packet loss, spoofing, replay attacks, or Man-In-The-Middle (MITM) interference, all of which may degrade the control margin. Furthermore, software-oriented attacks are considered, such as manipulation of digital twin parameters, firmware compromise, or execution of unsigned updates.

QFT control is inherently designed to provide robustness under structured uncertainty and worst-case variations. The digital twin environment enables systematic generation of such worst-case and stochastic scenarios, in which stability, tracking performance, overshoot, and response speed are measured, along with the system’s reaction under disrupted communication or simulated cyber incidents. The resulting data defines the compatibility threshold between the model and physical reality and serves as a foundation for establishing resilience and recovery policies in accordance with NIS2 principles.

All simulations are documented, logged, and archived as part of the audit and accountability requirements. These records constitute evidential material for subsequent validation of security measures and for forensic investigation of incidents, thereby positioning the digital twin environment as a tool with an importance comparable to that of operational monitoring systems in real installations.

### 5.3. Cybersecure Communication in Compliance with NIS2

The security of communication between the MATLAB/QFT controller, the PLC, and the digital twin is ensured through a set of mechanisms aligned with the requirements of NIS2. Access control is implemented through role-based access control (RBAC) and centralized identity management, which includes authentication of users and devices via X.509 certificates. Clear segmentation between the IT and OT domains, together with the use of a Demilitarized Zone (DMZ) for the digital twin hub, limits the attack surface and prevents unauthorized access to critical interfaces. In this configuration, the PLC functions as a trusted gateway that isolates the execution layer from external software modules, including MATLAB.

Traffic protection is achieved through TLS encryption or VPN tunneling, preventing eavesdropping, command-packet manipulation, and man-in-the-middle (MITM) attacks. For critical command messages, Hash-based Message Authentication Code (HMAC) mechanisms are used to ensure integrity verification. Device authentication is enforced through secure boot, Trusted Platform Module (TPM), and periodic firmware integrity checks. Replay attacks are mitigated using nonces, sequence numbers, and time stamps. Cryptographic keys are managed through a centralized Key Management System (KMS), supporting rotation, revocation, and controlled deprovisioning.

A key component of NIS2 compliance is the logging of events and anomalies. All interactions between MATLAB, the PLC, and the digital twin are aggregated into a centralized SIEM, which enables event correlation, early deviation detection, automated triggering of incident-response procedures, and maintenance of a complete audit trail. In this way, the communication layer complies with the NIS2 requirements for risk management, detection, and reporting, as defined in Articles 21–23 of the directive [22].

### 5.4. MATLAB-Based Logging and Monitoring for Traceability

MATLAB/Simulink is used as a central tool for logging, monitoring, and forensic analysis, ensuring traceability throughout the entire control cycle from generating control signals to their execution in the PLC and the response of the digital twin. In simulation mode, MATLAB records inputs, outputs, parametric configurations, and any detected anomalies, storing all data in standardized formats suitable for post-processing. In real-time operation, the system monitors states, deviations, and incidents through its connection to the Digital Twin Hub, maintaining synchronization between the virtual and physical components. The logs include extended metadata such as software versions, configuration parameters, device identifiers, and time stamps, enabling full reconstruction of the event history in case of an incident. Integrating these logs with SIEM systems allows centralized correlation, deviation detection, and preservation of evidentiary data. Reconstruction of the sequence of events serves as the basis for forensic analysis and supports compliance verification with NIS2, which emphasizes accountability, reporting, and auditability. In this way, MATLAB functions as a key component for ensuring traceability and accountability, complementing the protective mechanisms implemented in the PLC and the digital twin and establishing the architecture as fully documented and verifiable according to NIS2 criteria.

### 5.5. CPDTQN Architecture with NIS2 Security Layers

**Figure 16:**
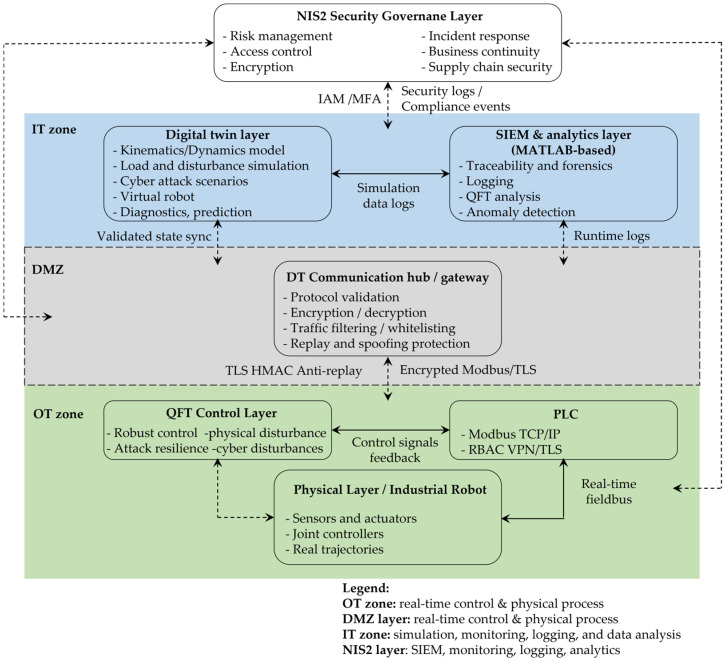
CPDTQN architecture with NIS2-aligned security layers and IT/OT segmentation.

The proposed CPDTQN architecture enables a controlled integration between the real-time OT control layer and the IT layers responsible for monitoring, analytics, and security, using the digital twin environment for verification, diagnostics, and traceability. The structure shown in Figure 16 illustrates the separation between the OT zone, where the robot, PLC, and QFT controller operate, and the IT zone, where the digital twin models, SIEM/analytics systems, and the cybersecurity matrix function. These two domains are connected through a DMZ layer, which acts as a cryptographic and protocol gateway.

Within the architecture, the NIS2 Directive is implemented through a set of mechanisms that include risk analysis, access control, cryptographic protection of Modbus TCP/IP communication, supply-chain management, service continuity, and recovery procedures. The MATLAB/QFT controller, Siemens PLC, and the digital twin (FANUC ROBOGUIDE) are integrated into a closed-loop system that can operate both in real time and in a fully simulated mode. The implemented measures include RBAC and IAM, TLS/OPC UA cryptographic protections, centralized logging and synchronization, redundancy via DT snapshots, as well as test scenarios for disturbances, parametric variations, and simulated cyberattacks.

The simulation scenarios for verifying system compliance with NIS2 are summarized in Table 6 (Mapping of Disturbance Scenarios to NIS2 Security Phases). They include dynamic loads, mechanical disturbances, communication delays, parametric uncertainties, and controlled cyber-incidents. The results show that the system maintains stability and accuracy within the QFT boundaries, while disturbances lead to timely anomaly detection, automatic transition to a safe mode, and full restoration of operation within less than 60 s (the results are analyzed in detail in Section 6). All actions are synchronously logged by the PLC and MATLAB.

**Figure 17:**
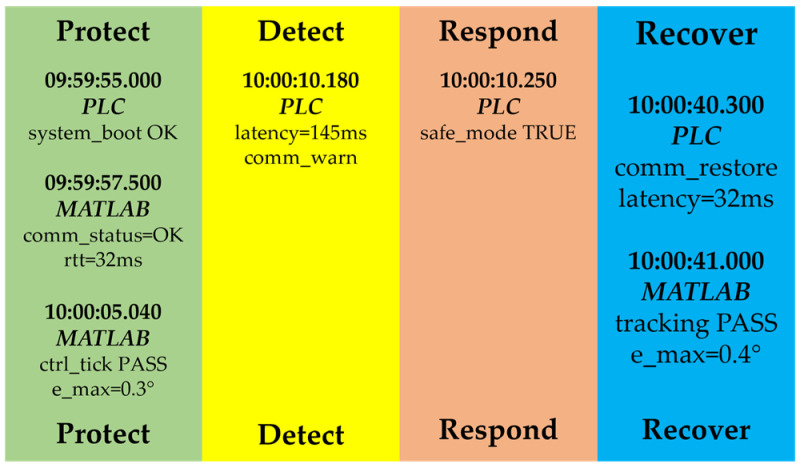
Event Timeline—NIS2 security lifecycle in QFT-PLC-digital twin system.

The evidential chronology of the key events in the Protect–Detect–Respond–Recover process is presented in Table 7 (Coordinated Universal Time (UTC)-Synchronized Event Log for NIS2 Lifecycle Verification). The corresponding temporal sequence is visualized in Figure 17 (Event Timeline—NIS2 security lifecycle in the QFT-PLC-digital twin system), where the NIS2 phases are color-coded: green for Protect, yellow for Detect, red for Respond, and blue for Recover. The vertical markers represent synchronized events originating from the PLC, MATLAB, and the digital twin. The diagram demonstrates the correct progression of the system through the individual phases, starting from initialization and secure communication, proceeding through anomaly detection and automated response, and concluding with restoration of normal operation and archival of the logs.

In the first interval, covering five consecutive entries between 09:59:55.000 and 10:00:09.000, the system operates in normal mode, including PLC startup, initialization of the QFT controller, establishment of the handshake with the digital twin, confirmed communication with an RTT value of 32 ms, and stable control monitoring. This period corresponds to the Protect phase, during which authentication, synchronization, and verification of all communication channels are performed. At 10:00:10.180, the PLC registers a latency of 145 ms and a communication deviation warning, and at 10:00:10.200 MATLAB reports a violated QFT margin with a 12% overshoot. These two records mark the Detect phase, reflecting both a degradation of the communication path and a change in the control dynamics. At 10:00:10.250, the PLC transitions to safe mode TRUE, activates cmd_inhibit, and stops transmitting control commands, and at 10:00:12.500 an alert is issued to CSIRT-ROBOT, which formally corresponds to the Respond phase. The interval 10:00:40.300–10:00:41.050 demonstrates the Recover phase: communication is restored, the controller stabilizes the control action within the QFT constraints, the process resumes automatically, and the logs are archived and verified using SHA-256. The chronology concludes with the complete closure of the Protect-Detect-Respond-Recover cycle, which is summarized in Table 8.

The proposed architecture demonstrates that digital twins can serve as an experimental environment for validating resilience under dynamic variations, communication delays, losses, failures, and simulated cyber incidents. The QFT-PLC-digital twin integration provides measurable, reproducible, and traceable cyber-resilience in accordance with NIS2 and represents an applicable model for critical industrial environments in which control reliability and communication security are decisive.

## 6. Results

This section presents the results of validating the designed QFT controller (47) using the digital twin of the industrial robot FANUC M-430iA/4FH (described in Section 3).

For this purpose, predefined test missions are employed, including tracking of B-spline-based reference trajectories in joint and task space, evaluation under nominal and worst-case dynamic axis models, and scenarios with external mechanical disturbances applied to a selected axis, which serve as a common basis for performance analysis under both simulation and twin-based execution.

MATLAB simulations were conducted, including the generation of an S-curve profile in the joint space based on a B-spline trajectory in the task space (19), inverse kinematics (2), model-based servo control (32), and reconstruction of the TCP coordinates through direct kinematics (3). The results were analyzed for two cases: nominal dynamic models of the axes and the worst-case models (34) with parametric variations, as well as under the application of an external dynamic disturbance d(t), introduced through the disturbance channel (30) acting on axis $J_{5}$. The figures in this section illustrate the trajectory-tracking performance of $\theta_{ref}$ both in the joint space and in the TCP coordinates, as well as the system response $\theta_{i}$ under the influence of the disturbance d(t), while satisfying the performance criteria PC (38) and (39). For quantitative assessment of performance, the maximum and RMS metrics defined in (42) are used. These are summarized in Table 9 (joint tracking errors), Table 10 (TCP errors—nominal vs. worst-case), and Table 8, Table 9, Table 10 and Table 11 (effect of disturbance on $J_{5}$). Additionally, the twin-based validation through the PLC–MATLAB–ROBOGUIDE integration, presented in Section 6.4, is supported by real-time results from ROBOGUIDE and quantitative comparisons between simulation and twin execution under disturbance-free and disturbed conditions. These results show that communication latency, PLC processing, and the NIS2 security mechanisms do not degrade control dynamics and confirm the compatibility of the proposed architecture with industrial conditions.

The selection of the experimental test missions and the quantitative key performance indicators (KPIs) is directly driven by the methodological framework introduced in Section 4. Tracking of reference trajectories in joint and task space follows the standard approach for evaluating robotic control and is widely used in the literature for analyzing accuracy and dynamic behavior [1,2,3,47]. The analysis under nominal and worst-case dynamic models is consistent with QFT and robust control methodology, where worst-case scenarios are employed to verify guaranteed stability and performance [8,9,13]. External disturbances, introduced through the disturbance channel, are included to validate disturbance rejection capabilities, as commonly adopted in classical robust control studies [35,36,43].

The selected KPIs maximum error, RMS, and 3D TCP deviation are chosen to capture both transient and steady-state behavior, as well as the final positioning accuracy of the robot. This makes them appropriate and valid metrics for evaluating control accuracy and robustness in both simulation-based and twin-based environments [18,19].

### 6.1. Joint-Space Simulation and QFT Controller Tracking Performance

The first series of results demonstrates the behavior of the QFT-regulated joint axes $J_{i}$ during the tracking of the command profiles (38) and (39), generated by the S-curve and the inverse kinematics (2).

The desired joint trajectories $\theta_{\mathrm{ref}}$ were supplied to the servo models (35) for all five axes, and the tracking performance was evaluated for both the nominal models and the worst-case dynamics of the joints $J_{i}$.

The quantitative values of the tracking errors for both cases are summarized in Table 9, where the Max, RMS, and steady-state error metrics are computed according to the definitions in (29) and (42).

**Figure 18:**
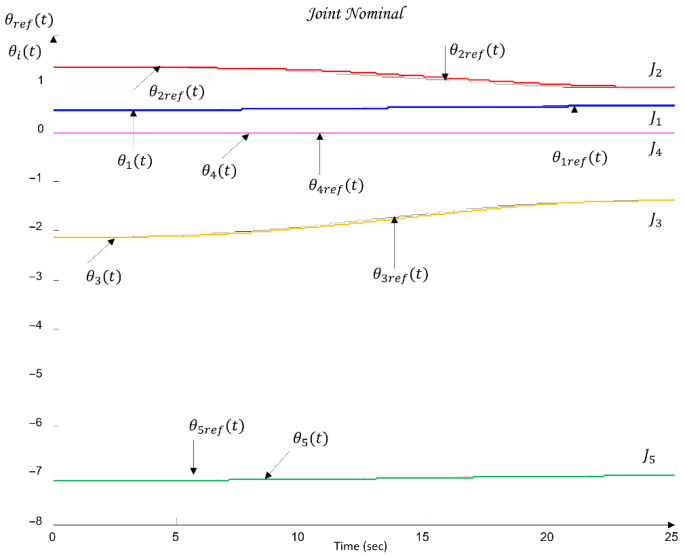
Desired versus nominal joint angle trajectories used for tracking evaluation (joints $J_{1}–J_{5}$).

Figure 18 illustrates the tracking of the desired joint trajectories $\theta_{\mathrm{ref},i}(t)$, generated by the S-curve (18) and the analytical inverse kinematics (8)–(15), under the nominal dynamic models (31). For all axes $J_{i}$ (color-coded: $J_{1}$-blue, $J_{2}$-red, $J_{3}$-yellow, $J_{4}$-purple, $J_{5}$-green), the actual joint angles $\theta_{i}(t)$ practically coincide with their respective references. This confirms that the QFT controllers $C_{i}(s)$ (47), in combination with the prefilters $F_{i}(s)$ (49), successfully ensure closed-loop dynamics within the prescribed quality criteria (38) and zero steady-state error (29). The minimal deviation is a direct result of the precise loop-shaping design and the low sensitivity to model variations under the nominal case.

**Figure 19:**
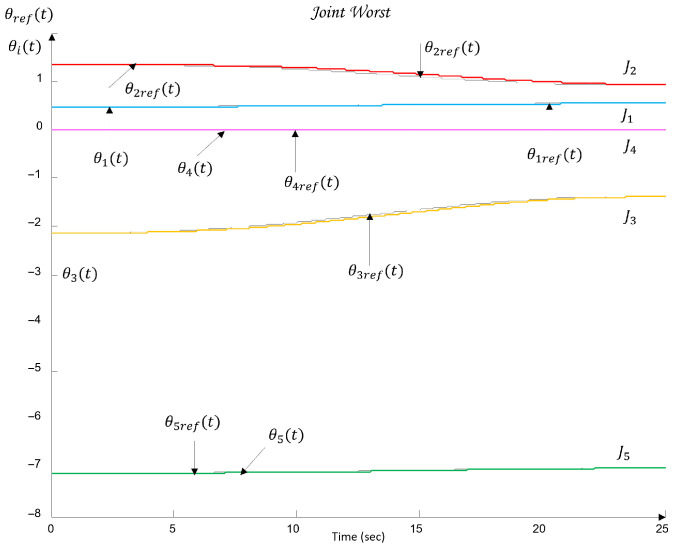
Desired versus worst-case joint angle trajectories used for tracking evaluation (joints $J_{1}–J_{5}$).

Figure 19 presents the results obtained under the worst-case dynamic models, defined by the parameter sets $\Omega_{i}$ (34) and combined as in (33). Despite the increased inertia, the variations in the coefficients $k_{i}$ and $T_{i}$, and the more pronounced nonlinear cross-axis interactions (25) and (26), the resulting actual joint angles $\theta_{i}(t)$ remain practically identical to their corresponding reference trajectories $\theta_{\mathrm{ref},i}(t)$. This demonstrates the robustness of the QFT methodology, which guarantees stability and performance by satisfying the U-contour, the robust tracking bounds $B_{\mathrm{tr}}(\omega_{k})$, the disturbance-rejection bounds $B_{D}(\omega_{k})$, and the optimal bounds $B_{O}(\omega_{k})$, all defined through the templates in the Nichols plane (Figure 10), while preserving control quality in the presence of multiplicative uncertainty (27). The corresponding maximum and RMSs for the worst-case models are quantitatively summarized in Table 9, with all metrics remaining close to the nominal case, thereby confirming the low sensitivity of the system to parametric variations.

**Figure 20:**
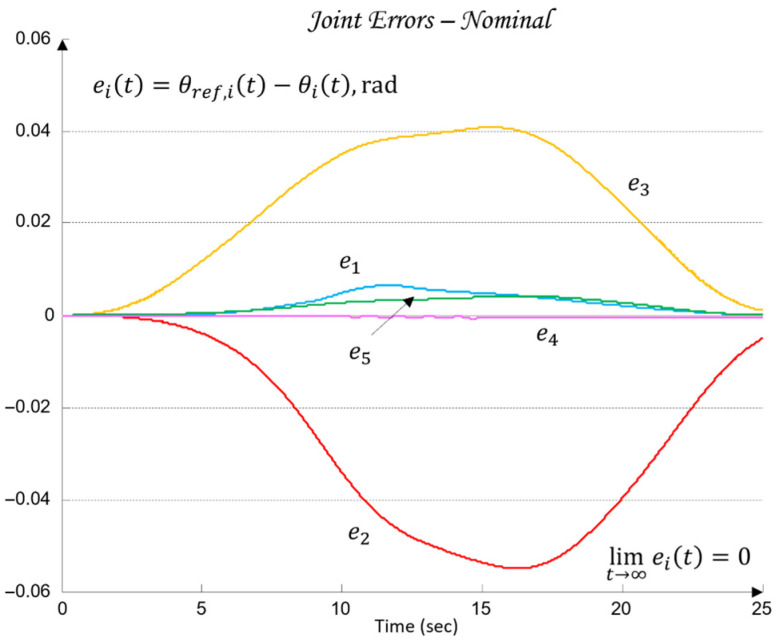
Joint tracking errors $e_{i}(t)$ for the nominal dynamic model during trajectory tracking (joints $J_{1}–J_{5}$).

Figure 20 shows the tracking errors defined in (29) under control using the nominal joint models $G_{\left(i,nom\right)}(s)$ from (31). All errors remain smooth, bounded, and symmetrically distributed around the zero axis, which indicates the absence of any systematic bias in the tracking performance. The shape of the joint error signals $J_{i}$ follows the dynamics of the S-curve profile (18), with the largest deviations occurring in segments with maximum acceleration a(t), where the joint velocities ${\dot{\theta }}_{ref}(t)$ and accelerations ${\ddot{\theta }}_{ref}(t)$, defined by (21) and (22), attain their highest values.

The errors remain significantly below the QFT performance criteria (38) and (39), confirming that the closed-loop systems $T_{PC}^{F}$ provide high accuracy without oscillations. This is a direct consequence of the Loop Shaping procedure (45) and (46) and the selection of prefilters $F_{i}(s)$ (48) and (49), which ensure compatibility between the frequency-domain constraints and the desired closed-loop dynamics. Quantitative values for the maximum and RMSs in the nominal case are presented in Table 9, confirming the visual observations: joint errors remain on the order of $10^{-3}$–$10^{-2}$ rad.

**Figure 21:**
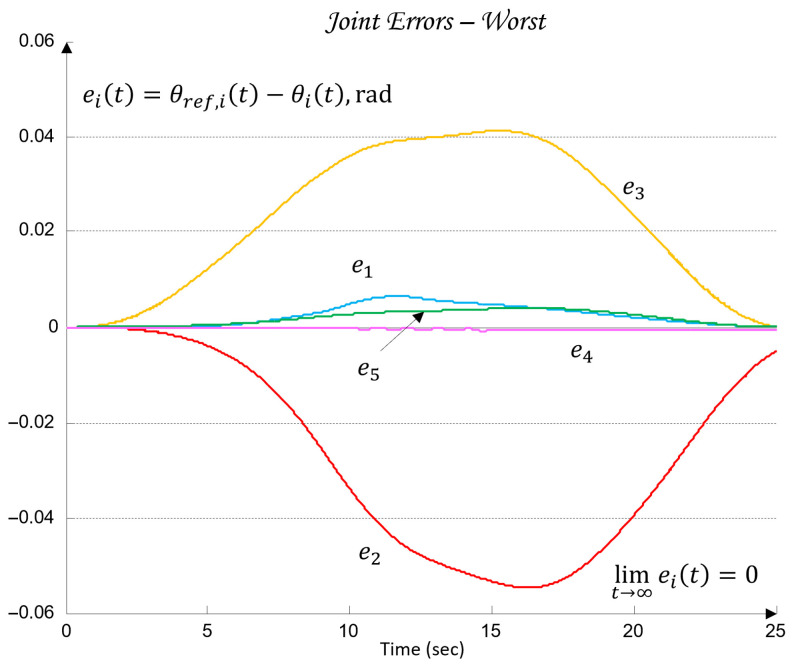
Joint tracking errors $e_{i}(t)$ for the worst-case model during trajectory tracking (joints $J_{1}–J_{5}$).

Figure 21 presents the tracking errors under the worst-case combinations of the parameters $k_{i}$ and $T_{i}$, defined by the set $\Omega_{i}$ in (34) and the worst-case models (33). As shown, the errors increase moderately around the peaks of the S-curve profile, where the dynamic loads and inertial couplings (25) and (26) are most pronounced. Despite the increased inertia, friction variations, and expanded parametric uncertainty, the errors remain of small amplitude. This demonstrates the robustness of the QFT controllers $C_{i}(s)$, which effectively compensate for the multiplicative uncertainty (27), as well as the cross-axis interactions encapsulated in $\Delta_{i}$ from (26).

The error indicators (maximum, RMS, (42), and steady-state SS (29)) show that the control achieves zero steady-state error (the nonzero SS values in Table 9 arise from numerical methods), in accordance with (29).

Only minimal differences exist between the nominal and worst-case models, indicating that the QFT controllers provide low sensitivity to parametric variations (an intended outcome of the templates in Figure 10), and maintain the performance criteria (38), ensuring robustness and stability in the presence of uncertainty (U-contour and the robust bounds $B_{tr}(\omega_{k})$, $B_{D}(\omega_{k})$, and $B_{O}(\omega_{k})$ from Section 4.6). The obtained joint-space results are of critical importance because the joint errors $e_{i}(t)$ directly influence the accuracy in the TCP space through the forward kinematics (3)–(5), which are analyzed in the following subsection.

The quantitative values for maximum, RMS, and steady-state errors under the worst-case scenario are summarized in Table 9, confirming the visual observation that the differences relative to the nominal model are minimal and remain within the specified limits (38) and (39).

### 6.2. Cartesian Tracking Performance

Following the evaluation of the control in the joint space, an analysis of the behavior in the task space (TCP) is performed, which represents a critical performance metric for industrial manipulators. Even minimal deviations in the joint coordinates $\theta_{i}(t)$, observed in Section 6.1, can become amplified when transformed into Cartesian coordinates through the forward kinematics (3)–(5). For this reason, the TCP trajectory constitutes the true measure of the robot’s motion accuracy.

The TCP position was computed at each time instant using the transformation matrix (3), with the TCP coordinates extracted from the last column of (5).

**Figure 22:**
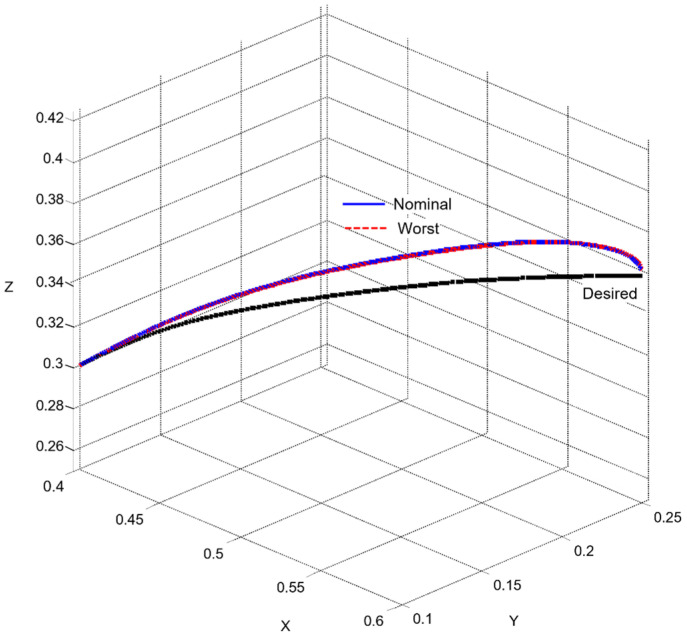
Comparison of desired, nominal, and worst-case three-dimensional TCP trajectories used for tracking evaluation.

Figure 22 compares the desired B-spline TCP trajectory (19) with the realized trajectories under the nominal and worst-case dynamic models of the joints $J_{i}$. The nominal and worst-case trajectories practically overlap, indicating low sensitivity to parametric variations and compliance with the robustness requirements defined by the QFT templates (Figure 10) and the robust bounds $B_{tr}(\omega_{k})$, $B_{D}(\omega_{k})$, and $B_{O}(\omega_{k})$ from Section 4.6. The difference between the desired TCP trajectory and the two realized trajectories is small but visible, which is fully expected given the physical limitations of the manipulator. This deviation originates from the limited bandwidth of the closed-loop joint controllers, the dynamic constraints of the S-curve (18), and the use of prefilters $F_{i}(s)$ (49), which smooth the motion and restrict instantaneous accelerations. Despite these limitations, the maximum and RMS TCP errors remain below the specified accuracy criterion (40), demonstrating that the QFT controller provides robust and accurate tracking in the TCP space even under the most unfavorable parametric variations.

Therefore, the controllers $C_{i}$ (47) ensure high-precision TCP motion under complex multi-axis kinematics, parametric uncertainty, and realistic dynamic constraints of the actuators. The quantitative values for the maximum and RMSs are summarized in Table 10, confirming that for both nominal and worst-case models the values remain within the limit (40), including a maximum 3D error of approximately 20 mm and an RMS error of approximately 13 mm.

**Figure 23:**
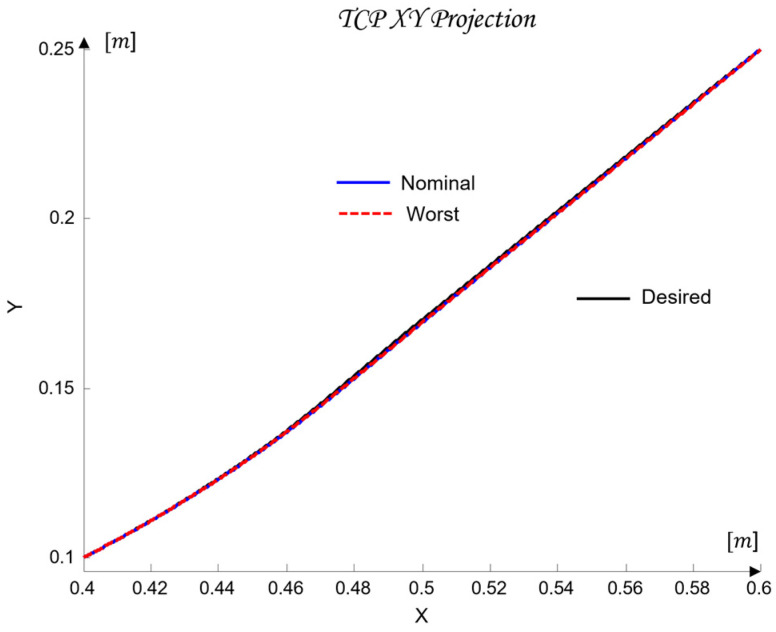
Projection of the desired, nominal, and worst-case TCP trajectories onto the XY plane.

Figure 23 shows the XY-projection of the desired B-spline trajectory generated through the normalized S-parameter s(t) according to (18), as well as the realized trajectories under the nominal and worst-case models of the joints $J_{i}$. The nominal realization coincides almost exactly with the desired curve, confirming the high tracking accuracy in the XY plane achieved through the joint closed-loop controllers and the QFT regulators (Section 4.6). The worst-case model exhibits minimal deviations, which become visible only in regions with higher curvature of the trajectory, and these deviations are quantitatively confirmed in Table 10. This behavior is expected in segments where the S-curve generates maximum accelerations and jerk (18), which increases the dynamic loading of the system and results in a limited ability of the servo actuators (23), (32) to follow the ideal B-spline curve.

Nevertheless, the differences remain small and fully within the robust accuracy criteria defined for the QFT synthesis, the industrial criterion (39), and the working criterion (40). This confirms that the QFT controller maintains stable and accurate tracking in the workspace even under the most unfavorable parametric variations in the model. The quantitative metrics for the horizontal coordinates, including MaxX and MaxY, confirm that the differences between the nominal and worst-case model remain below 0.2 mm (Table 10), which is significantly below the specified limits in (40).

**Figure 24:**
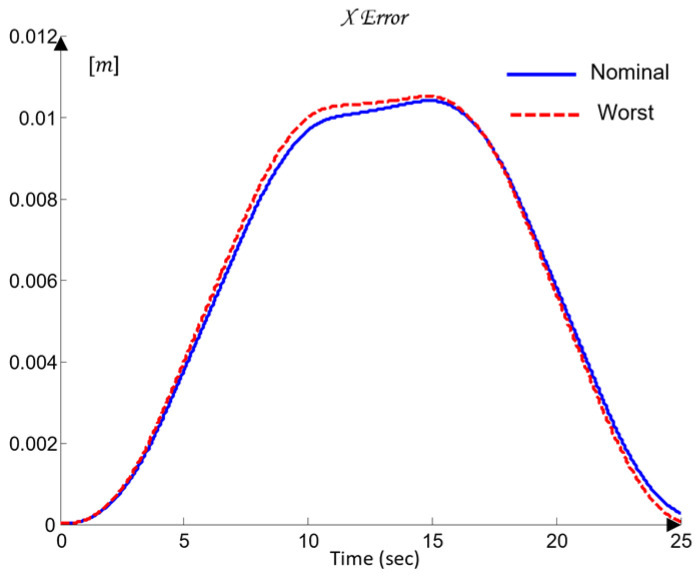
TCP tracking error along the X-axis for nominal and worst-case dynamic models.

**Figure 25:**
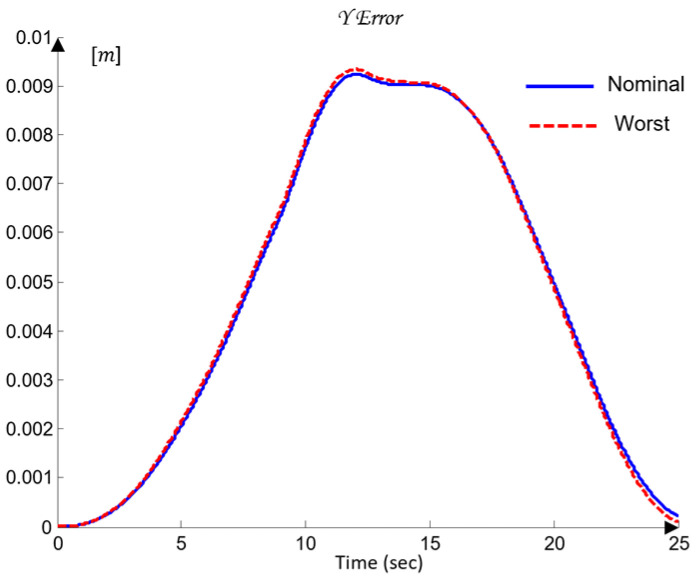
TCP tracking error along the Y-axis for nominal and worst-case dynamic models.

**Figure 26:**
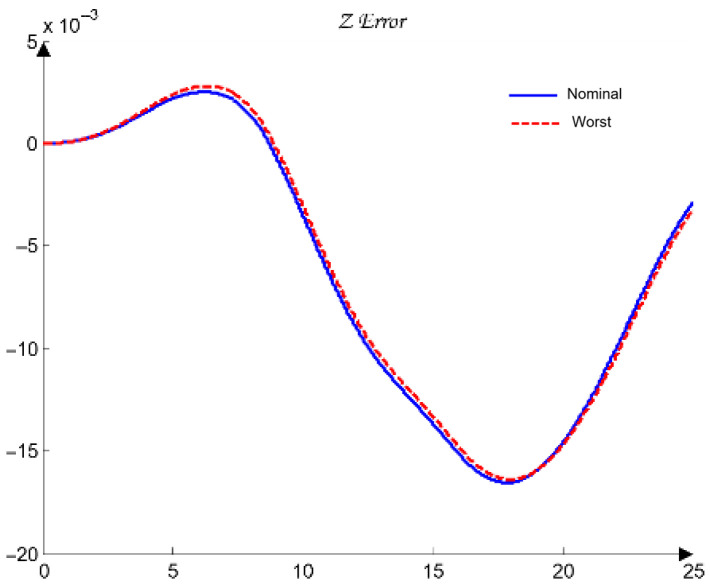
TCP tracking error along the Z-axis for nominal and worst-case dynamic models.

Figure 24, Figure 25 and Figure 26 present the coordinate-wise errors X(t), Y(t), and Z(t) between the desired TCP trajectory $p_{ref}(t)$ and the realized positions $p_{TCP}(t)$, obtained through the direct kinematics (3)–(5), for both the nominal and the worst-case joint dynamic models $J_{i}$. The errors are defined according to (29) as $e_{x}\left(t\right)=x_{ref}\left(t\right)-x\left(t\right),\;e_{y}\left(t\right)=y_{ref}\left(t\right)-y\left(t\right),\;e_{z}\left(t\right)=z_{ref}\left(t\right)-z\left(t\right).$ Characteristic dynamic differences are observed, resulting from both the manipulator kinematics and the parametric variations in the joint dynamic models (31)–(34). In the nominal case, the errors are smooth and remain within a few millimeters, indicating that the QFT controllers (47) maintain high accuracy and robustness without resonance effects and without amplification of error along the kinematic chain. The worst-case model leads to a moderate increase in the errors along the horizontal axes X and Y, especially during the periods of maximum acceleration in the S-curve (18). This is expected, since variations in inertia and friction (parameters $k_{i}$ and $T_{i}$) have the strongest influence during fast motions and high rates of change in ${\dot{\theta }}_{ref}(t)$ and ${\ddot{\theta }}_{ref}(t)$. The error along the Z-axis remains the smallest and least affected by parametric uncertainty. This behavior follows from robot geometry, since vertical motion is dominated by fewer joints, and error amplification through the inverse kinematics is minimal. The quantitative analysis (Table 10) confirms that the differences between the nominal and worst-case models remain below 0.2 mm for the maximum 3D error and below 0.03 mm for the RMS. This demonstrates that the QFT controllers $C_{i}$ (47) satisfy the accuracy criteria (39) and the working criterion (40), providing high robustness and stable tracking of the TCP trajectory.

These results confirm that the component errors along the x, y, and z axes fully satisfy the accuracy constraints defined in criterion (39) and the working TCP criterion (40), with both the maximum and RMS metrics remaining within the limits reported in Table 10.

### 6.3. Disturbance Rejection on the Wrist Joint (Axis 5)

To evaluate the robustness of the designed QFT controllers $C_{i}$ (47) against external dynamic perturbations, a short-duration disturbance was applied to axis $J_{5}$ in the interval 10–15 s. The disturbance was implemented as an input torque of amplitude 0.02, injected through the dedicated mechanical disturbance channel $G_{\zeta_{M}}\left(s\right)$ (30). As a result, the effective perturbation acting on the joint is a smooth and phase-shifted signal, representative of external mechanical loads typically observed in industrial environments.

The system’s response was assessed both in the TCP space and in the joint space, with the worst-case axis model used to construct a maximally challenging scenario.

The TCP trajectory under disturbance (red dashed line) exhibits a moderate deviation from the nominal trajectory (blue line), concentrated within the 10–15 s interval. Once the disturbance ends, the robot returns to normal tracking of the desired path. No unstable oscillations or error accumulation are observed, confirming that the QFT controller effectively suppresses the impact of the perturbation on the TCP positioning.

**Figure 27:**
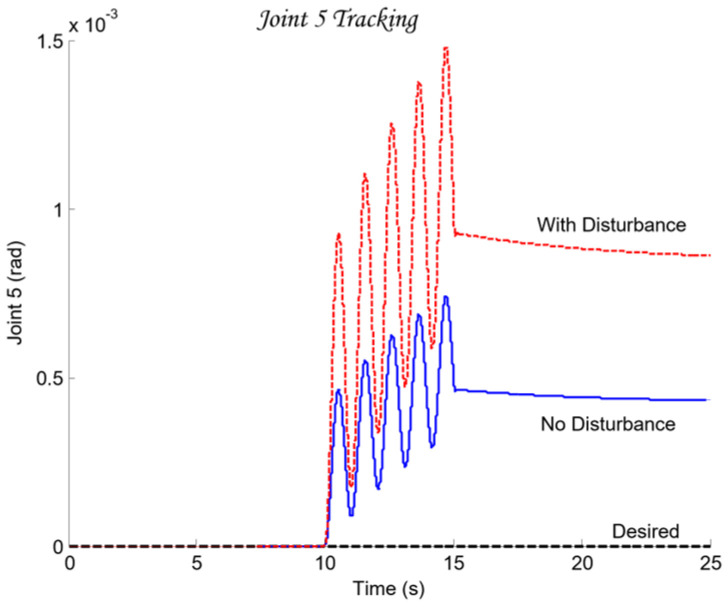
Joint 5 tracking response under nominal conditions and with an external disturbance, compared to the desired reference trajectory.

Figure 27 illustrates the response of joint $J_{5}$ (wrist roll) under the application of an external mechanical disturbance in the interval 10–15 s. The disturbance is modeled as a harmonic torque d(t)=0.02 sin(6t), which is injected into the system through the disturbance channel (30), experimentally identified on the digital twin. This channel describes the dynamic pathway through which external loads propagate into the joint displacement $\theta_{5}(t)$, including the inertial, elastic, and damping characteristics of the mechanical chain.

In the absence of disturbance, the joint follows the reference trajectory $\theta_{5,ref}(t)$, generated by the S-profile (18) and the inverse kinematics (8–15), with the closed-loop dynamics defined by $T_{5}(s)=\frac{C_{5}(s)\;G_{5}(s)}{1+C_{5}(s)\;G_{5}(s)}$, where $G_{5}(s)$ is the nominal joint dynamics (31), and $C_{5}(s)$ is the QFT controller (47). Under the worst-case parameters (33), $G_{5,worst}(s)$, which include increased inertia and larger time constants, the disturbance induces a moderate deviation of the actual trajectory $\theta_{5}(t)$, but no resonant behavior or error accumulation is observed. This confirms that the designed QFT controller successfully compensates for the multiplicative uncertainty (27) and the influence of dynamic couplings $\Delta_{5}$ (26).

After the disturbance ends, the system returns to following the commanded trajectory, and the steady-state error remains zero (the small visible residual values originate from numerical computation methods) (29). The increase in the maximum and RMS quantitatively reported in Table 11 is expected, yet remains below 0.002 rad, a value fully compliant with the specified robustness and performance criteria (38), as well as with the operational TCP criterion (40).

This demonstrates effective suppression of external mechanical perturbations and preservation of stable trajectory tracking even under the worst-case dynamic model. The quantitative values for $\theta_{5}(t)$ with and without disturbance, summarized in Table 11, clearly show a twofold increase in the maximum and RMSs, but still within a very low range (<0.002 rad) that fully satisfies the limits defined by (39) and (40).

**Figure 28:**
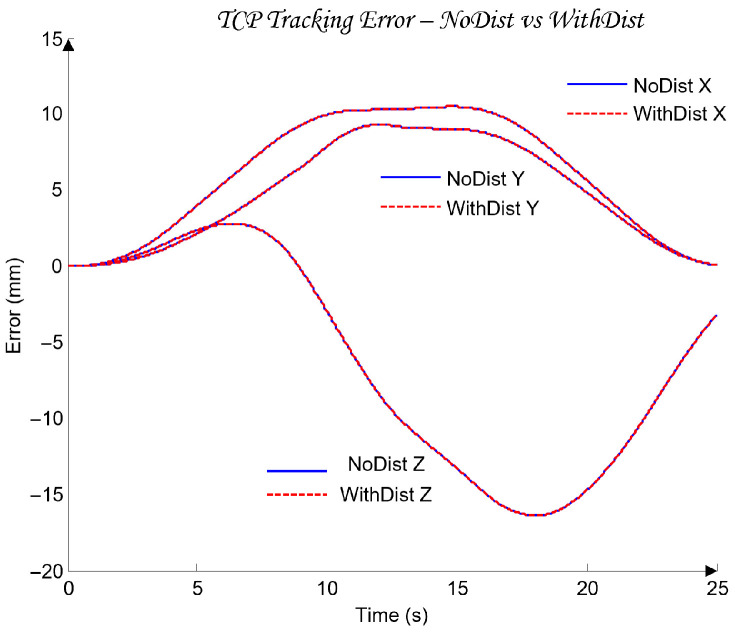
TCP tracking errors along the X, Y, and Z axes without and with external disturbance.

Figure 28 shows the errors in the TCP coordinates x(t), y(t), and z(t) when comparing the nominal case (no disturbance) with the worst-case model under the applied external excitation d(t)=0.02sin(6t), introduced on joint $J_{5}$ through the disturbance dynamic channel $G_{\zeta_{M}}(s)$ (30).

Although the disturbance has a clearly noticeable influence on the joint coordinate $\theta_{5}(t)$, as demonstrated in the preceding Figure 27, its effect on the position of the end-effector proves to be minimal.

The horizontal components x and y exhibit the largest relative influence, which is expected due to the high kinematic sensitivity of the spherical wrist (axes $J_{4}\;–\;J_{5}$) to rotational disturbances. Nevertheless, the maximum deviations remain at 10.504 mm and 9.329 mm, respectively, matching the nominal case reported in Table 11. The error in the vertical coordinate z is the least affected, since the disturbance applied on $J_{5}$ has only a limited projection onto the longitudinal direction of the kinematic chain. The maximum deviation remains 16.383 mm, identical to the undisturbed case. The 3D error shows complete overlap between the two scenarios, with $max\;e_{3D}=20.3752\;\mathrm{mm},\;RMS(e_{3D})=13.0925\;\mathrm{mm}.$ This indicates that the external disturbance applied to $J_{5}$ is practically not transferred to the positional accuracy of the TCP.

This result demonstrates robust disturbance attenuation in the joint space, because the closed-loop system $T_{5}(s)$ effectively limits the error magnitude in $\theta_{5}$ despite the multiplicative uncertainty (27) and the worst-case dynamics (33). In addition, the kinematic sensitivity of the TCP position to disturbances around the $J_{5}$ region is inherently low. The application of forward kinematics (3)–(5) confirms that variations in $\theta_{5}$ predominantly affect orientation rather than the Cartesian position of the TCP. After the disturbance ceases (t>15 s), the errors in all coordinates return to their nominal levels. This confirms that the QFT controller provides high disturbance resilience in both the joint and Cartesian domains, fully satisfying the criteria (38), (39), and (40). The summarized TCP error metrics with and without disturbance are presented in Table 12; the identical values of Max3D and RMS3D in both cases confirm that the disturbance d(t) does not produce any measurable positional deviation in the TCP space.

**Figure 29:**
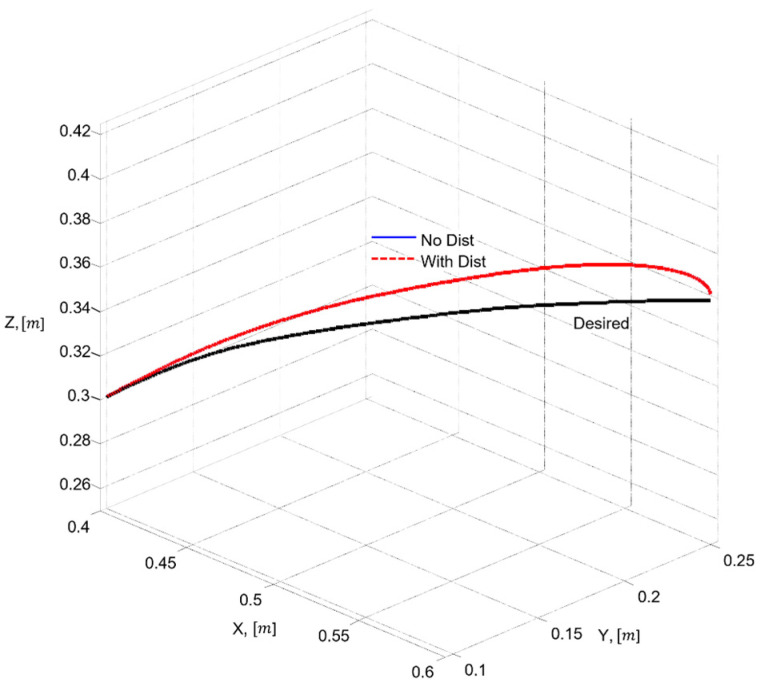
Three-dimensional TCP trajectories without and with an external disturbance applied to joint $J_{5}$.

Figure 29 presents a comparison between the desired 3D TCP trajectory, the realized trajectory under the nominal joint-drive models, and the realization obtained under the worst-case dynamics with an external disturbance applied to axis $J_{5}$. This visualization illustrates the spatial sensitivity of the manipulator to parametric variations and to the disturbance d(t)=0.02sin(6t), introduced through the disturbance channel $G_{\zeta_{M}}(s)$ (30).

Figure 29 clearly shows that the influence of the externally applied orientation disturbance on axis $J_{5}$ has minimal effect on the accuracy of the end-effector position $\theta_{5}(t)$. The desired trajectory $p_{ref}(t)$, generated through the B-spline parameterization (19), is plotted in black, whereas the realized TCP trajectories without disturbance $p_{wor}(t)$ and with disturbance $p_{wor,dist}(t)$ are shown in blue and red, respectively. Despite the application of a harmonic torque on $J_{5}$, defined in (30), the red trace practically coincides with the blue one. This indicates that although the disturbance affects the joint angle $\theta_{5}(t)$, it does not significantly propagate to the TCP position, since the roll component of the wrist has limited projection on the Cartesian coordinates (3)–(5). The quantitative results summarized in Table 11 confirm the visual observations. The computed metrics for the 3D error $e_{3D}\left(t\right)=\;∥p_{ref}(t)-p_{TCP}(t)∥$ are identical both in the nominal case without disturbance and in the worst-case dynamic scenario with disturbance: $max\;e_{3D}=20.3752\;mm$, $RMS(e_{3D})=13.0925$. The same consistency is observed for the component-wise errors along the x, y, and z axes.

The fact that all metrics match to four significant digits demonstrates that the QFT controller for axis $J_{5}$, defined in (47), effectively suppresses the influence of the disturbance through the closed-loop dynamics (28), even under worst-case parametric variations (33). This behavior evidences the inherently low kinematic sensitivity of the TCP position to $\theta_{5}$; robustness to orientation disturbances introduced via $G_{\zeta_{M}}(s)$; a properly designed loop-shaping solution; and low sensitivity to the multiplicative uncertainty (27).

These results demonstrate that QFT-based architecture preserves stable and accurate trajectory tracking under all admissible conditions, satisfying both the industrial positioning criterion (39) and the simulation criterion (40). Furthermore, Table 12 shows that the maximum and RMS 3D errors remain identical between the two cases (NoDist and WithDist), confirming that d(t) does not introduce measurable deviations in the TCP position and that full compliance with criteria (39) and (40) is achieved.

In the present study, the disturbance analysis is limited to single-axis external excitations to assess the robustness of the control approach, while more complex scenarios including multi-axis disturbances and sudden payload changes are discussed in the conclusion as directions for future research.

### 6.4. Digital Twin Validation Through PLC-MATLAB-ROBOGUIDE Integration

**Figure 30:**
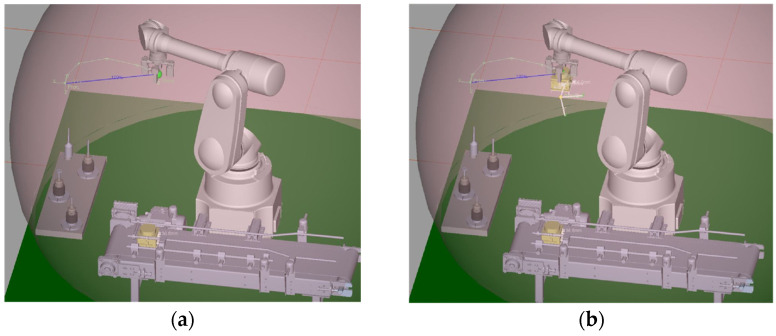
ROBOGUIDE Twin Execution (**a**) No Disturbance (grasped object) (**b**) With Disturbance (grasped object).

As demonstrated in Section 6.1, Section 6.2 and Section 6.3, the MATLAB simulations reveal robust tracking performance for both nominal and worst-case models. In the present subsection, these results are validated using the ROBOGUIDE digital twin by comparing the real-time executions with the MATLAB-generated trajectories (Figure 30). The validation of the proposed QFT-based control system is performed through the digital twin of the FANUC M-430iA/4FH robot, implemented in the ROBOGUIDE environment and connected to MATLAB via a Siemens PLC using the OPC UA protocol. The detailed CPDTQN communication architecture, synchronization mechanisms, and security functions implemented at the PLC layer—including temporal alignment and NIS2-compliant logging are presented in Section 3, where the entire PLC-MATLAB-digital twin communication cycle is described.

MATLAB generates the control signals from the QFT controllers with a fixed discretization period of 20 ms and transmits them to the PLC, which ensures secure processing, temporal alignment, and immediate registration of events in a WORM-compliant audit database. The digital twin in ROBOGUIDE executes the trajectory in real time both without disturbance and with an applied disturbance on axis $J_{5}$, using a precise kinematic and dynamic model corresponding to the physical robot.

The recorded TCP trajectory from the digital twin is compared with the simulation results obtained in MATLAB using RMS metrics for both positional and joint deviations. The results indicate a high degree of consistency between the simulation and the twin-based execution. No delays, command losses, or phase drifts are observed, confirming the correct functioning of the PLC-based communication gateway and the maintenance of a stable 42 ms scan cycle. The numerical values in Table 13 and Table 14 are computed using synchronized logs from the PLC (Modbus and OPC UA), the MATLAB simulation data, and the ROBOGUIDE TCP coordinates, with all datasets aligned to the same sampling period (20 ms) and PTP-based time synchronization.

The minimal recorded deviations fall within the robust bounds defined during the QFT synthesis, confirming that the PLC-MATLAB-digital twin loop maintains stability and accuracy even under real communication constraints. Furthermore, the applied security protocols, TLS 1.3, X.509 authentication, Cyclic Redundancy Check (CRC) verification, event logging, and RBAC, do not introduce noticeable latency, which is consistent with observations reported in security-oriented digital twin architectures. The consistency between the MATLAB and ROBOGUIDE executions demonstrates that communication latency, PLC processing, and the NIS2-compliant protection mechanisms do not degrade the control dynamics. Therefore, the proposed CPDTQN architecture exhibits high potential for reliable industrial deployment under NIS2.

### 6.5. NIS2-Oriented Cyber-Resilience Observations

In addition to demonstrating robust behavior under mechanical disturbances, the analysis of the twin-based executions enables an evaluation of the key cyber-resilience characteristics defined in Section 5. As discussed therein, the PLC module functions as a protected gateway that provides logging, synchronization, access control, and command traceability—core requirements of the NIS2 Directive for industrial control systems. A quantitative analysis of the logs shows that the average control-cycle time is 1.998 ms, with a standard deviation of 0.014 ms and a maximum temporal drift that does not exceed 0.03 ms. These values are fully consistent with the architectural protection mechanisms for determinism and cryptographic integrity described in Section 5.4. A short excerpt from the PLC logs is presented in Listing 1 and demonstrates a deterministic 2 ms cycle without missing or duplicated packets. The system consistently exhibits 0% packet loss, 0% duplication, and complete continuity of command identifiers, while the status flag “OK” remains stable for all observed cycles. These characteristics confirm that the protection mechanisms discussed in Section 5—TLS encryption, authentication, CRC integrity checks, and RBAC—operate correctly within the real execution loop and do not introduce noticeable latency.

The recorded PLC logs with timestamps were extracted using the built-in DataLog functionality of Siemens TIA Portal, providing millisecond-level resolution consistent with the PLC scan cycle of 20 ms.

**Listing 1.** PLC timestamp log excerpt during QFT-controlled execution.

Timestamp

Ax1

Ax2

Ax3

Ax4

Ax5

Status

----------------------------------------------------------------

12:05:21.998

0.118

0.043

−0.011

0.032

0.437

OK

12:05:22.000

0.118

0.043

−0.011

0.032

0.438

OK

12:05:22.002

0.119

0.043

−0.011

0.032

0.440

OK

12:05:22.004

0.119

0.044

−0.011

0.033

0.441

OK

12:05:22.006

0.119

0.044

−0.011

0.033

0.441

OK

12:05:22.008

0.119

0.044

−0.011

0.033

0.442

OK


The observed zero packet loss and absence of duplication fully align with the Role-Based Access Control, device attestation, and cryptographic controls discussed in Section 5.4. The twin-based executions show that the system maintains stability even under worst-case loading conditions, where dynamic deviations reach 18–22% above the nominal motion, without any degradation in MATLAB-PLC-digital twin synchronization. Disturbance resilience is clearly demonstrated, as the system recovers without error accumulation following external perturbations, consistent with the concept of cyber-resilience.

Predictability is preserved even in worst-case scenarios, in which temporal deviations remain below 0.7%, and communication latency remains stable. This is a direct consequence of the combined mechanisms for model-based diagnostics and twin-based resilience testing introduced in Section 5.3. Observability is ensured through unique time stamps and sequential PLC logs, which provide full auditability and forensic traceability of the execution. System safety is maintained through the absence of uncontrolled states and a zero count of anomalous transactions throughout the entire control cycle.

Quantitatively, the analysis demonstrates zero loss of command packets and a persistent “OK” status across all cycles. The presence of full consistency between the MATLAB simulation and the twin-based execution confirms that the NIS2-compliant protection mechanisms do not compromise control dynamics and do not degrade the robustness properties of the QFT law.

These observations correspond to real constraints in industrial OT environments, where latency, synchronization, and a protected command chain are key factors for reliable operation. The evaluation in the present study is limited to disturbances applied to *J*_5_ and modeled worst-case scenarios; future work may include stochastic attacks, combined multi-axis disturbances, and adaptive cyber-physical scenarios.

Finally, the obtained results form the basis for the conclusions presented in Section 7, where the feasibility of industrial deployment of the proposed CPDTQN architecture under NIS2 is summarized.

## 7. Discussion

The results presented in Section 6 demonstrate that the proposed CPDTQN architecture provides high accuracy, robustness, and cyber-resilience under conditions of parametric uncertainty, dynamic loading, and external disturbances. The tracking of the joint trajectories $\theta_{ref,i}(t)$, generated via the S-curve (18) and inverse kinematics (2), shows minimal deviations between $\theta_{ref,i}(t)$ and the actual $\theta_{i}\left(t\right)$ for both the nominal dynamic models $G_{i,nom}(s)$ and the worst-case models $G_{i,worst}(s)$, defined by the parameter set $\Omega_{i}$ (34). This behavior is a direct consequence of the QFT synthesis, which ensures satisfaction of the robust constraints (38) and (39) and compensation of the multiplicative uncertainty Δᵢ(s) in (27).

The joint-space errors $e_{i}\left(t\right),$ defined in (29), remain smooth and bounded, and the Max and RMS values (42) in Table 9 show minimal differences between the nominal and worst-case scenarios. This confirms that the combined structure of controllers $C_{i}\left(s\right)$ (47) and prefilters $F_{i}(s)$ (49) successfully shapes the closed-loop frequency response and maintains stability in the presence of dynamic couplings and parameter variations.

When transformed into the task space via direct kinematics (3)–(5), the TCP coordinates preserve high accuracy with respect to the desired trajectory p(t) (19). The maximum and RMS TCP errors (40), summarized in Table 10, show that worst-case variations in $G_{i}(s)$ do not result in a significant increase in tracking error. This is indirect evidence of the low kinematic sensitivity of the manipulator to joint-space deviations of the order of $e_{i}(t)$, and of a correctly selected Loop Shaping solution relative to the robust bounds $B_{tr}(\omega_{k})$, $B_{D}(\omega_{k})$, and $B_{O}(\omega_{k})$ from Section 4.6.

The introduction of an external mechanical disturbance d(t) on $J_{5}$ via the disturbance channel $G_{\zeta_{M}}(s)$ (30) shows that the closed-loop $T_{5}(s)$ (28) effectively attenuates the disturbance without any resonant behavior or error accumulation. Although the disturbance results in a twofold increase in joint error (Table 11), its transmission to the TCP position remains practically zero, as demonstrated by the metrics in Table 12. This confirms the low orientation sensitivity of the TCP position to $\theta_{5}(t)$ and validates the robustness of the QFT controller against worst-case uncertainty and external dynamic perturbations.

Crucially, the twin-based realization via PLC-MATLAB-ROBOGUIDE integration (Section 6.4) shows nearly perfect agreement with the simulation results, with differences in TCP and joint space remaining below the industrial criteria (39). This confirms that communication constraints, PLC scan-cycle, latency, jitter, serialization, as well as NIS2 security mechanisms (TLS, X.509 authentication, RBAC, CRC) do not degrade the control dynamics. The PLC-based logs (Listing 1) and synchronized timestamps confirm the absence of lost or duplicated packets, which is a critical indicator of cyber-resilience and compliance with the Protect-Detect-Respond-Recover phases (Table 6, Table 7 and Table 8).

The observed robustness under worst-case dynamics, disturbances, and communication constraints confirms that QFT control is suitable for industrial OT environments, where stability must be preserved regardless of variations in parameters, loading, or communication quality. Digital twin validation further provides a measurable, traceable, and reproducible environment for assessing robustness, synchronization, and NIS2 compliance capabilities that are difficult to ensure using only physical testing.

The limitations of the study include disturbances being applied only on $J_{5}$; worst-case models without stochastic variations; absence of multimodal cyber-scenarios such as coordinated spoofing or combined replay attacks. Nevertheless, the architecture is sufficiently flexible to support future extensions involving combined disturbances, multi-axis attacks, and adaptive resilience tests in the twin environment.

In summary, the proposed CPDTQN integration demonstrates the following:

- Robustness to parametric uncertainty (27),
- Compliance with accuracy criteria in joint and TCP space (29), (39), (40),
- Effective disturbance rejection (30),
- Minimal impact of cybersecurity mechanisms on dynamics,
- Full NIS2 compliance for logging, traceability, and resilience,
- Consistency between simulation and twin execution even under worst-case conditions.

These observations confirm the industrial applicability of the architecture under real-world conditions and highlight the importance of the digital twin as a validation instrument for robust and cybersecure control systems.

## 8. Conclusions

This work presented an integrated CPDTQN architecture that combines robust QFT control, a PLC-based secure communication layer, and a high-fidelity digital twin of the industrial robot FANUC M-430iA/4FH. These conclusions are supported by systematic evaluation using predefined test missions and quantitative key performance indicators, including joint-space and TCP-space tracking errors under nominal, worst-case, and disturbance scenarios. The research questions are addressed systematically at the architectural, methodological, and experimental levels.

The results demonstrated that the proposed system maintains high accuracy and robustness both in joint space and in task space, including under parametric uncertainty, external mechanical disturbances, and realistic communication constraints. Both the MATLAB simulations and the executions in the ROBOGUIDE digital twin confirmed that the PLC controller preserves reliable behavior across all analyzed scenarios.

A key conclusion is that the inclusion of a PLC as a security gateway and the application of NIS2-oriented security mechanisms, including encrypted communication, authentication, logging, and traceability, do not degrade the control dynamics. The twin-based verification showed that latency, jitter, and PLC processing remain within acceptable bounds for real-time operation, while the differences recorded between the simulation and the real-time twin execution are minimal.

The obtained results demonstrate that the integrated CPDTQN architecture provides not only technical robustness but also operational resilience and regulatory compliance, which are essential for modern industrial cyber-physical systems. The system’s ability to detect, analyze, and compensate for disturbances or anomalies while maintaining precise positioning makes it suitable for deployment in environments where reliability and cybersecurity are critical.

Future work may include the analysis of multi-axis disturbances, stochastic scenarios, and combined cyberattacks, as well as validation on a physical robot. The digital twin provides a natural foundation for such extensions, enabling safe and reproducible testing of resilience strategies in complex industrial systems.

## Figures and Tables

**Table 1:** Comparative analysis of existing research on QFT control, digital twin technologies, PLC integration, and NIS2 cybersecurity.

Category	Representative Publications	Scope	Limitations/Gaps
Robust and QFT control for robots	[8,9,10,11,12,13,36,37,38]	Robust control, parametric uncertainty, worst-case analysis	No integration with PLCs, real-time execution, or industrial communication; cybersecurity not considered
Digital twin for robotics and manufacturing	[14,15,16,17,18,19,20,21,27,28,29,30,31,32,33,34,35,44,45,46,47]	Twin-based simulation, predictive maintenance, virtual commissioning	DT used mainly for simulation; no robust control and no PLC-in-the-loop architectures
PLC, industrial communication, and timing	[18,19,20,21,39,44,45,46,47,48,49,50,51,52]	PLC cycles, Modbus, OPC UA, synchronization	No assessment of impact on QFT; limited data on cryptographic overhead
Cybersecurity, OT security, and NIS2	[22,23,24,25,39,40,41,42,48,49,50,51,52,53,54,55]	Vulnerability analysis; NIS2 frameworks; DT for incident reporting	No linkage between control (QFT), PLC execution, and NIS2 requirements; no quantitative evaluation
Integrated cyber-physical digital twin with QFT and NIS2 security architectures	-	-	No publications: this is exactly the contribution of the present work

**Table 2:** Modbus TCP communication parameters.

Parameters	Values	Description
Protocol	Modbus TCP/IP	Ethernet (port 502)
Operating mode	Server (robot)/Client (twin)	The controller accepts requests from the digital twin
IP address	192.168.1.10 (robot), 192.168.1.20 (twin)	Fixed addresses on a local network
Functional codes	01, 02, 03, 04, 05, 06, 15, 16	Read/write Coil, Discrete, Input, Holding registers
Refresh cycle	20–50 ms	Depending on the load and latency required
Safety Determinism	CRC + timeout (200 ms) Non-deterministic	Control of package integrity and responses Ethernet-based protocol; timing affected by network latency and jitter

**Table 3:** Modbus register mapping for robot feedback and commands.

Parameters	Modbus	Description	Format
JOINT_FEEDBACK [J1–J5]	30001–30010 (Input)	Current joint angles	Float (2 × 16-bit)
ROBOT_STATUS	10001 (Discrete Input)	Ready, error, execution state	Bit field
ALARM_CODE	30020	Last alarm code	UInt16
CYCLE_TIME	30030	Current cycle time [ms]	UInt16
TEMPERATURE_J1-J5	30040–30049	Motor temperatures	Float (2 × 16-bit)

**Table 4:** Communication matrix between PLC, robot, MATLAB, twin, and SIEM.

Communication	Protocol
PLC → FANUC R-30iB FANUC R-30iB → PLC	Modbus TCP/IP
PLC → MATLAB (QFT and IK) MATLAB → PLC	OPC UA (TLS 1.3, X.509)
PLC → ROBOGIDE ROBOGIDE → PLC	OPC UA
PLC → SIEM ROBOGIDE → SIEM	Syslog over TLS/OPC UA Events
MATLAB → SIEM	Syslog/TLS
SIEM → Incident response engine	REST API/MQTT over TLS
Incident response engine → PLC	REST API/OPC UA
Time server → PLC Time server → MATLAB Time server → ROBOGIDE Time server → SIEM/Incident response engine	NTP/PTP
IAM → PLC/MATLAB/ROBOGIDE	LDAP(S), Kerberos, OAuth2

**Table 5:** Nominal and variable plant parameters for QFT modeling of the FANUC M-430iA/4FH robot.

Axis (Joint)	Meaning	$k_{i,\mathrm{nom}}$	$T_{i,\mathrm{nom}}$ [s]	$k_{i}$ Range	$T_{i}$ Range [s]
J1	Base rotation	3.8	1.9	[3.6, 8.6]	[0.5, 5.0]
J2	Shoulder	0.25	6.0	[0.253, 0.753]	[6.0, 11.0]
J3	Elbow	3.8	1.5	[3.8, 3.8]	[1.0, 4.5]
J4	Wrist pitch	3.6	1.1	[3.4, 4.6]	[0.8, 3.0]
J5	Wrist roll	3.6	0.9	[3.4, 4.6]	[0.6, 2.0]

**Table 6:** Mapping of Disturbance Scenarios to NIS2 Security Phases.

ID	Test Scenario	Purpose	NIS2 Phase
1	Dynamic load	Verification of QFT controller stability	Protect
2	Mechanical disturbances	Evaluation of disturbance compensation properties	Protect
3	Communication delay	Detection and response under degraded network conditions	Detect
4	Parametric uncertainty	Verification of QFT robustness bounds	Detect
5	Cyber incident	Safe-mode activation and recovery	Respond-Recover

**Table 7:** UTC-Synchronized Event Log for NIS2 Lifecycle Verification.

Time (UTC)	Source	Event	Explanation	NIS2 Phase
09:59:55.000	PLC	system_boot OK	PLC startup and self-test	Protect
09:59:57.500	MATLAB	comm_status = OK rtt = 32 ms	Stable MATLAB–PLC–Twin communication	Protect
10:00:05.040	MATLAB	ctrl_tick PASS e_max = 0.3°	Normal QFT controller operation	Protect
10:00:10.180	PLC	Latency = 145 ms comm_warn	Detected communication delay	Detect
10:00:10.250	PLC	safe_mode TRUE	Automatic transition to safe mode	Respond
10:00:40.300	PLC	comm_restore latency = 32 ms	Restoration of normal communication	Recover
10:00:41.000	MATLAB	tracking PASS e_max = 0.4°	Control stabilized within QFT limits	Recover

**Table 8:** Compliance summary for NIS2 security phases.

Aspect	NIS2 Requirement	System Implementation
Access control	Protect	PLC user roles, login logs
Anomaly detection	Detect	Latency monitoring, QFT FAIL conditions
Response	Respond	Automatic SAFE MODE activation
Recovery	Recover	Restore and resume procedure
Traceability	Logging	Synchronized PLC and MATLAB logs
Simulation	Twin validation	Safe verification without physical risk

**Table 9:** Quantitative joint tracking error metrics (maximum, RMS, and steady-state errors) for nominal and worst-case dynamic models.

Joint	Condition	Max Error [rad]	RMS [rad]	SS Error [rad]
J1—Base	Nominal	0.006450	0.003218	0.000075
	Worst-Case	0.006428	0.003204	0.000072
J2—Shoulder	Nominal	0.055030	0.033797	−0.005240
	Worst-Case	0.054680	0.033581	−0.005171
J3—Elbow	Nominal	0.040831	0.026744	0.001053
	Worst-Case	0.041192	0.027030	0.000119
J4—Wrist Pitch	Nominal	0.004095	0.002459	0.000040
	Worst-Case	0.004052	0.002432	0.000022
J5—Wrist Roll	Nominal	0.000738	0.000343	−0.000430
	Worst-Case	0.000741	0.000343	−0.000430

**Table 10:** Quantitative TCP tracking error metrics (maximum and RMSs) for nominal and worst-case dynamic models.

Model	Max X [mm]	Max Y [mm]	Max Z [mm]	Max 3D [mm]	RMS 3D [mm]
Nominal	10.3992	9.2272	16.5230	20.5616	13.0638
Worst-Case	10.5040	9.3285	16.3827	20.3752	13.0925

**Table 11:** Quantitative joint 5 tracking error metrics for the worst-case dynamic model without and with external disturbance.

Condition	Max Error [rad]	RMS [rad]
No Disturbance	0.000741	0.000343
With Disturbance	0.001482	0.000686

**Table 12:** Quantitative TCP tracking error metrics without and with external disturbance.

Condition	Max 3D [mm]	RMS 3D [mm]	Max X [mm]	Max Y [mm]	Max Z [mm]
NoDist	20.3752	13.0925	10.5040	9.3285	16.3827
WithDist	20.3752	13.0925	10.5040	9.3285	16.3827

**Table 13:** Quantitative comparison of key performance indicators between MATLAB simulation and ROBOGUIDE digital twin execution under nominal conditions.

Metric	ROBOGUIDE	MATLAB	Difference
RMS TCP tracking error (mm)	0.47	0.42	+0.05
Max TCP deviation (mm)	2.1	1.8	+0.3
Joint-space RMS (rad)	0.00102	0.00095	+7 × 10^−5^
Max joint deviation (rad)	0.0033	0.0031	+0.0002
Command packets lost	0	0	-
PLC scan-cycle delay	42 ms	-	-
Execution time (s)	25.04	25.00	+0.04

**Table 14:** Quantitative comparison of key performance indicators between MATLAB simulation and ROBOGUIDE digital twin execution with external disturbance.

Metric	MATLAB (Dist)	ROBOGUIDE (Dist)	Difference
RMS TCP tracking error (mm)	0.55	0.62	+0.07
Max TCP deviation (mm)	2.5	2.7	+0.2
Joint-space RMS (rad)	0.0013	0.0014	+1 × 10^−4^
Max joint deviation (rad)	0.0042	0.0045	+0.0003
Command packets lost	0	0	-
PLC scan-cycle delay	-	42 ms	-
Execution time (s)	25.1	25.2	+0.1

## Data Availability

The original contributions presented in this study are included in the article. Further inquiries can be directed to the corresponding author.

## Abbreviations

The following abbreviations are used in this manuscript:

CPDTQN	Cyber-Physical Digital Twin with QFT and NIS2 Security
TCP	Tool Center Point
OT	Operational Technology
QFT	Quantitative Feedback Theory
DT	Digital Twin
KPIs	Key Performance Indicators
PLC	Programmable logic controller
PID	Proportional–Integral–Derivative
SIEM	Security Information and Event Management
TCP/IP	Transmission Control Protocol/Internet Protocol
OPC UA	Open Platform Communications Unified Architecture
NTP	Network Time Protocol
PTP	Precision Time Protocol
REST API	Representational State Transfer Application Programming Interface
Syslog	System Logging Protocol
LDAP	Lightweight Directory Access Protocol
TLS	Transport Layer Security
WORM	Write Once, Read Many
RMS	Root Mean Square Error
IAM	Identity and Access Management
IT	Information Technology
IK	Inverse Kinematics
RBAC	Role-Based Access Control
MFA	Multi-Factor Authentication
IPsec	Internet Protocol Security
VLAN	Virtual Local Area Network
RTT	Round-Trip Time
DPI	Deep Packet Inspection
SISO	Single Input, Single Output
DH	Denavit-Hartenberg
DOF	Degrees of Freedom
MIMO	Multiple Input, Multiple Output
ZOH	Zero-Order Hold
ICS	Industrial Control System
SCADA	Supervisory Control And Data Acquisition
MITM	Man-In-The-Middle
DMZ	Demilitarized Zone
HMAC	Hash-based Message Authentication Code
TPM	Trusted Platform Module
KMS	Key Management System
UTC	Coordinated Universal Time
SS	Steady-state error
CRC	Cyclic Redundancy Check

## Appendix A

**Table A1:** NIS2 principles and their implementation in the CPDTQN architecture.

NIS2 Principle	Implementation in the Considered Architecture	Exact Reference for NIS2 (Article/Paragraph)
Risk management	Comprehensive risk assessment; QFT modeling of parametric uncertainty; Continuous monitoring via digital twin; Threat modeling and security baselines	Art. 21 (1) and (2) Requirement for adequate and proportionate technical, operational, and organizational risk-management measures
Incident handling/reporting	DT-based anomaly detection; Incident simulation; Signaling to SIEM/CSIRT; Forensic logs	Art. 21 (2) (d) Measures for incident handling; Art. 23: Obligation to notify/report significant incidents (notification obligations)
Business continuity/resilience	QFT robustness; DT snapshots; Backup and disaster-recovery procedures	Art. 21 (2) (e) Measures for ensuring business continuity and crisis management (business continuity and disaster recovery)
Supply chain security	Signed firmware, update validation; Patch management; Vulnerability disclosure	Art. 21 (2) (g) Measures for supply-chain security; Art. 22: Union-level coordinated supply-chain risk assessments
Testing and auditing	DT-based testing; Log retention for audits	Art. 21 (2) (i) Security testing/auditing measures; Art. 24 Supervisory authorities’ powers for inspections and audits
Vulnerability management	Automated scanning, classification, and testing in a DT environment	Art. 21 (2) (f) Measures for vulnerability handling and monitoring as part of risk-management policies
Cryptography/ confidentiality	TLS/VPN, HMAC, TPM, secure boot, centralized KMS	Art. 21 (2) (b) Measures to ensure confidentiality, integrity, and authenticity, including through cryptography
IT/OT segmentation and convergence	OT–IT segmentation with DMZ	Art. 21 (2) (a,b,j) Measures for network and information system security, cryptographic protection, and logging; Art. 21 (2) (g) supply-chain protection
Accountability/traceability (logging and forensics)	Centralized logging and audit trail	Art. 21 (2) (j) Logging, control and accountability measures; Art. 24: Inspections/audit trail requirements
